# PMCA inhibition reverses drug resistance in clinically refractory cancer patient-derived models

**DOI:** 10.1186/s12916-023-02727-8

**Published:** 2023-02-01

**Authors:** Ki Cheong Park, Jung Min Kim, Sang Yong Kim, Seok-Mo Kim, Jin Hong Lim, Min Ki Kim, Sungsoon Fang, Yonjung Kim, Gordon B. Mills, Sung Hoon Noh, Jae-Ho Cheong

**Affiliations:** 1 Department of Surgery, Systems Cancer Biology & Biomarker Research Lab, Yonsei University College of Medicine, Seoul, Republic of Korea; 2 Severance Biomedical Science Institute, BK21 PLUS project for Medical Science, Yonsei University College of Medicine, Seoul, Republic of Korea; 3 EONE-DIAGNOMICS Genome Center, New drug R&D Center, 291 Harmony-ro, Yeonsu-gu, Incheon, 22014 Republic of Korea; 4 Department of Systems Biology, the University of Texas MD Anderson Cancer Center, Houston, TX USA; 5 Brain Korea 21 Project for Medical Science, Yonsei University College of Medicine, Seoul, Republic of Korea; 6 YUMC-KRIBB Medical Convergence Research Institute, Yonsei University College of Medicine, Seoul, Republic of Korea; 7 Department of Biochemistry & Molecular Biology, Systems Cancer Biology & Biomarker Research Lab, Yonsei University College of Medicine, Seoul, Republic of Korea

**Keywords:** Glucose deprivation-induced metabolic stress-resistant cancer, Drug-resistant cancer, PGC1α, PMCA, Candidate 13

## Abstract

**Background:**

Cancer cells have developed molecular strategies to cope with evolutionary stressors in the dynamic tumor microenvironment. Peroxisome proliferator-activated receptor-γ coactivator-1α (PGC1α) is a metabolic rheostat that regulates diverse cellular adaptive behaviors, including growth and survival. However, the mechanistic role of PGC1α in regulating cancer cell viability under metabolic and genotoxic stress remains elusive.

**Methods:**

We investigated the PGC1α-mediated survival mechanisms in metabolic stress (i.e., glucose deprivation-induced metabolic stress condition)-resistant cancer cells. We established glucose deprivation-induced metabolic stress-resistant cells (selected cells) from parental tumor cells and silenced or overexpressed PGC1α in selected and parental tumor cells.

**Results:**

Several in vitro and in vivo mouse experiments were conducted to elucidate the contribution of PGC1α to cell viability in metabolic stress conditions. Interestingly, in the mouse xenograft model of patient-derived drug-resistant cancer cells, each group treated with an anti-cancer drug alone showed no drastic effects, whereas a group that was co-administered an anti-cancer drug and a specific PMCA inhibitor (caloxin or candidate 13) showed marked tumor shrinkage.

**Conclusions:**

Our results suggest that PGC1α is a key regulator of anti-apoptosis in metabolic and genotoxic stress-resistant cells, inducing PMCA expression and allowing survival in glucose-deprived conditions. We have discovered a novel therapeutic target candidate that could be employed for the treatment of patients with refractory cancers.

**Supplementary Information:**

The online version contains supplementary material available at 10.1186/s12916-023-02727-8.

## Background

Nutrient deprivation is a critical physiological stressor with a fatal impact on cell viability. To cope with a lack of nutrients, cancer stem-like cells (CSCs) have developed various molecular strategies, including metabolic reprogramming to protect energy balance and cell survival, resulting in the acquisition of survival mechanisms under glucose deprivation-induced metabolic stress conditions compared with non-CSCs [1–3]. The evolutionary pressure due to the tumor microenvironment results in the selection of clones that can survive in metabolically challenging microenvironments. Metabolic reprogramming induced by nutrient starvation can be mediated by several molecular players [4, 5]. Among these, peroxisome proliferator-activated receptor-γ coactivator 1α (PGC1α) is a metabolic rheostat that controls energy homeostasis by regulating oxidative metabolism [6, 7]. Recently, Wolf et al. reported that the expression of PGC1α is upregulated, and mitochondrial oxidative phosphorylation is subsequently increased in drug-resistant subpopulations of tumor cells and CSCs [8, 9], suggesting a common molecular strategy shared by highly malignant cancer subtypes in metabolic and genotoxic contexts.

Glucose deprivation causes endoplasmic reticulum (ER) stress by interfering with N-linked protein glycosylation [10, 11]. ER stress is an important adaptive cellular process, activated in response to a range of diverse stimuli and pathological conditions, which triggers evolutionarily conserved molecular responses [12]. The importance of cellular cytosolic free calcium signaling in cell death and survival has previously been studied in response to ER stress [13, 14]. ER stress can cause free calcium release from ER stores into the cytoplasm; the subsequent accumulation of free calcium in the mitochondrial matrix can temporarily activate oxidative phosphorylation [15]. However, if prolonged, mitochondrial apoptotic signals are triggered, amplified, and executed when cytosolic free calcium concentration is elevated beyond physiological levels [16]. Therefore, although it is an adaptive response to glucose deprivation, sustained high cytosolic calcium concentration in ER stress exerts detrimental effects on cell survival [11].

Plasma membrane calcium ATPases (PMCAs) are key players in the regulation of cytosolic free calcium concentration in cells and thus essential to cytoprotection through the efficient extrusion of overloaded free calcium to outside cells [17]. Calcium is involved in several types of cell death and plays a crucial role in making cellular life-or-death decisions [18, 19]. Physiologically, PMCA is regulated by free calcium and plays an important role in mediating calcium signaling, including calcium-mediated cell death [20]. As cancer cells reprogram their metabolic process to protect their energy homeostasis and promote cell survival, there is a significant breach in calcium-mediated apoptosis [21, 22], which results in a universal increase in survival capacity. In particular, this augmented survival capacity could be characteristic of a subpopulation of tumor cells that survive the ever-changing metabolic microenvironment, implying a connection between metabolic sub-clonal selection and malignant tumor progression.

In this study, glucose deprivation-induced metabolic stress-selected MDA-MB231 (S-231) and MCF-7 cells showed significantly increased PMCA expression compared with non-surviving cancer cells (parent cells: P-231 and P-MCF-7).

Notably, our research found that PMCA was regulated by PGC1α. Knockdown of PGC1α expression in S-231 cells significantly decreased PMCA levels, leading to apoptosis during prolonged glucose deprivation. These data provide evidence that PGC1α is a key regulator of anti-apoptosis in selected cancer cells by inducing PMCA expression, thereby allowing survival in metabolically detrimental conditions. Furthermore, the inhibition of PMCA in combination with 2-deoxy-d-glucose (2DG) treatment, which mimics glucose deprivation, in a mouse tumor xenograft model, provides insights into a potential novel therapeutic strategy for the treatment of malignant subtypes of tumors selected in tumor metabolic environments.

## Methods

### Patient characteristics

YUMC-C1, YUMC-C2, YUMC-P1, and YUMC-M1 cells were obtained from tumor specimens of patients after their last surgery. Further details are as described in Additional file 1: Supplementary Methods.

### Patient tissue specimens

Fresh tumor samples were collected from patients with biochemically and histologically established recurrent cancer with metastasis, who were treated at the Severance Hospital, Yonsei University College of Medicine, Seoul, Korea. Fresh tumor samples were obtained during surgical resection.

### Ethical considerations

The research protocol was approved by the Institutional Review Board of Severance Hospital, Yonsei University College of Medicine (Institutional Review Board Protocol: 3-2019-0281). Cell samples were obtained from patients at the Severance Hospital, Yonsei University College of Medicine, Seoul, South Korea.

### Tumor cell isolation and primary culture

After resection, tumor tissue samples were maintained in normal saline supplemented with antifungal and antibiotic agents and transferred to the laboratory. Normal tissue and fat were removed and the tumor tissues were rinsed with 1× Hank’s Balanced Salt Solution. Further protocol details are as previously published [23].

### Cell culture

The patient-derived cancer cells, MDA-MB-231 and MCF-7 cells, were obtained from fresh tumors of patients or ATCC (ATCC, Manassas, VA, USA). Further details are described in Additional file 1: Supplementary Methods.

### Cell viability assay

Cell viability assay was measured using 3-(4,5-dimethylthiazol-2-yl)-2,5-diphenyltetrazolium bromide (MTT) assay according to the manufacturer’s protocol. We measured the viability of selected cells after treatment with caloxin (PMCA inhibitor), nifedipine, or verapamil (both calcium channel blockers) under glucose-deprived conditions.

### mRNA sequencing and analysis

Total RNA was isolated from the patient-derived cancer cells, MDA-MB-231 and MCF-7 cells, using TRIzol reagent (Thermo Fisher Scientific, Waltham, MA, USA) according to the manufacturer’s instructions. Further details are described in Additional file 1: Supplementary Methods.

### Hierarchical clustering

Hierarchical clustering analysis was performed using complete linkage and Euclidean distance as a measure of similarity to display the expression patterns of differentially expressed transcripts that were satisfied with |fold change|≥2 and independent *t*-test raw *p* < 0.05. All data analysis and visualization of differentially expressed genes were conducted using R 3.5.1.

### Protein–protein interaction (PPI)

PPI was analyzed based on the STRING database and visualized using Cytoscape. PPI was sorted by combined score (combined score ≥ 0.4 was considered the threshold value) and connecting total gene counts (>3 counts). The combined score is computed by combining the probabilities from the different evidence channels and corrected for the probability of randomly observing an interaction.

### Total RNA extraction and quantitative reverse transcription-polymerase chain reaction (qRT-PCR)

Total RNA was prepared from tumor cells by extraction using the RNeasy Mini Kit (Qiagen, cat. 74106) and One-Step RT-PCR Kit (Qiagen, # 204243) according to the manufacturer’s protocols. All data were normalized to *α-tubulin* expression. Primers for *PMCA1*, *PMCA2*, *PMCA3*, and *PMCA4* (Additional file 2: Supplementary Table 1) were used for performing qRT-PCR.

### Intracellular calcium measurements by microspectrofluorimetry

The intracellular Ca^2+^ level of MB231 and MCF-7 cells were imaged using a calcium-sensitive fluorescent dye, Fura-2AM. Cells were incubated with Fura-2AM in normal PBS solution for 30 min at 37 °C, followed by de-esterification of the dye for another 30 min at room temperature (22–25°C). Fura-2AM was excited at a wavelength of 340 nm, and emitted light was filtered with a 380-nm band pass filter. Fluorescence intensities (∆*F*) were normalized to the resting values. Cells were perfused with 140 mM NaCl, 5.4 mM KCl, 2 mM CaCl2, 1 mM MgCl2, 33 mM glucose, and 20 mM HEPES (pH 7.4, adjusted with NaOH, and 320–350 Osm with sucrose).

### Immunofluorescence analysis and confocal imaging

The expression levels of PMCA and PGC1α were analyzed by immunofluorescence staining. Cells cultured on glass-bottomed dishes (MatTek, Ashland, MA, USA) were fixed with 4% formaldehyde solution (R&D Systems, Abingdon, UK) for 10 min and permeabilized with 0.5% Triton X-100 in phosphate-buffered saline (PBS) for 10 min. The slides were air dried, washed with PBS, and incubated overnight at 4 °C with anti-PMCA (1:50, Abcam, #2825) and anti-PGC1α (1:25; Abcam, Cambridge, UK, #106814) in 3% bovine serum albumin which included PBS. After being washed with PBS, slides were incubated with Alexa 488 (1:200; Molecular Probes, Eugene, OR, USA) for 1–2 h at room temperature. Nuclei were stained with Hoechst 33342 (Life Technologies) for visualization. Images were observed under a confocal microscope (LSM Meta 700; Zeiss, Oberkochen, Germany) and analyzed using the Zeiss LSM Image Browser, version 4.2.0121.

### Subcellular fractionation and immunoblot analysis

Antibodies against caspase-3 (1:200, #56053), caspase-7 (1:200, #81654), CHOP (1:100, #7351), Histone H3 (1:200, #18521), and β-actin (Santa Cruz Biotechnology, CA, USA, #47778), PMCA (1:500, #2825), and PGC1α (1:500, Abcam, Cambridge, UK, #54481) were used. Equal amounts of protein were separated on 8–10% sodium dodecyl sulfate-polyacrylamide gels; the resolved proteins were electro-transferred onto polyvinylidene fluoride membranes (Millipore, Bedford, MA, USA). The membranes were subsequently blocked with 5–10% nonfat milk in TBST for 1 h at room temperature or overnight at 4 °C and incubated with appropriate concentrations of primary antibodies overnight at 4 °C. The membranes were then rinsed 3–10 times with TBST and probed with the corresponding secondary antibodies conjugated to horse radish peroxidase (Santa Cruz) at room temperature for 1 h. After rinsing, the blots were developed with ECL reagents (Pierce) and exposed using Kodak X-OMAT AR Film (Eastman Kodak, Rochester, NY, USA) for 1–5 min. Nuclear fractions were prepared using the NE-PER Nuclear and Cytoplasmic Extraction reagents (Thermo Fisher Scientific; #78833) in accordance with the manufacturer’s instructions. Separated nuclear and cytoplasmic extracts were isolated with a protein extraction solution (PRO-PREP, iNtRON Biotechnology, Seoul, Korea, #17081) or histone extraction kit (Abcam, Cambridge, UK, #113476). Protein extracts and bands were quantified using NanoDrop 2000 (Thermo Fisher Scientific, Waltham, MA, USA) and ImageJ software (NIH, Bethesda, MD, USA).

### Cell lines, stable transfection of siPGC1α, and overexpression of PGC1α

The CSC MDA-MB231 and MCF-7 cells were transfected with *PGC1α* small interfering RNA (siRNA) or control scrambled siRNA, according to the manufacturer’s protocol. The sequence of the *PGC1α* siRNA and methods are described in Additional file 2: Supplementary Table 2 and Additional file 1: Supplementary Methods.

### Electrophoretic Mobility Shift Assay (EMSA)

The DNA binding activity of hepatocyte nuclear factor 4 α (HNF4α) and nuclear factor (NF) κB against PMCA1 and 2 promoters was confirmed using a ^32^P-labeled oligonucleotide. Specific labeled and unlabeled oligonucleotides are described in Additional file 2: Supplementary Table 3 and Additional file 1: Supplementary Methods.

### Dual-luciferase assays

Promoter activity was evaluated using the dual-luciferase reporter assay (Promega, Madison, WI, USA; E1960), according to the manufacturer’s protocol. Regions of HNF4α- and NFκB-binding sites were amplified via PCR using human genomic DNA as a template. Further details are described in Additional file 1: Supplementary Methods and previously published [24].

### Pharmacophore- and docking-based sequential virtual screening for the identification of a novel PMCA inhibitor

Molecular analysis and virtual screening were performed by Cowell Biodigm Co., Ltd. In brief, after preparing the structure of PMCA, a pharmacophore-based virtual screening approach was applied to 230 million commercially available compounds using the ZINC database [25]. The pharmacophore model was generated, and the compounds matching the model were searched using BIOVIA Discovery Studio 2020. The selected 37,490 compounds were subjected to molecular modeling studies with the CDOCKER module [26] of DS software. Further details are described in Additional file 1: Fig. S1.

### Immunohistochemistry

Immunohistochemical staining was performed using a standard protocol. Primary monoclonal antibodies against PMCA1, PMCA2, and PMCA-pan were diluted with phosphate-buffered saline at a ratio of 1:50. All tissue sections were counterstained with hematoxylin, dehydrated, and mounted. Further protocol details are described in Additional file 1: Supplementary Methods and published previously [24].

### Image analysis

MetaMorph 4.6 software (Molecular Devices, San Jose, CA, USA) was used for the computerized quantification of immunostained target proteins.

### In vivo mouse xenograft study

Cancer cells obtained from ATCC (MDA-MB-231 and MCF-7 cells; 1.0×10^6^ cells/mouse) and cancer cells isolated from patients (YUMC-C1, YUMC-C2, and YUMC-P1 cells; 4.5×10^6^ cells/mouse) were cultured in vitro and then injected subcutaneously into the upper left flank region of 6-week-old female BALB/c nude and NOD/Shi-scid, IL-2Rγ KOJic (NOG) mice. Further details are described in Additional file 1: Supplementary Methods. Animals were maintained under specific pathogen-free conditions, and all experiments were approved by the Animal Experiment Committee of Yonsei University (2020-0002).

### Statistical analysis

Statistical analyses were performed using GraphPad Prism 6.0 (GraphPad Software, La Jolla, CA, USA), and the immunohistochemistry results were evaluated via analysis of variance followed by the Bonferroni post hoc test. Values are expressed as the mean ± standard deviation, and *p* < 0.05 indicates statistical significance.

## Results

### Distinct gene expression and signaling activation between metabolic stress-resistant and non-resistant cancer cells under glucose deprivation-induced metabolic stress

**Fig. 1:**
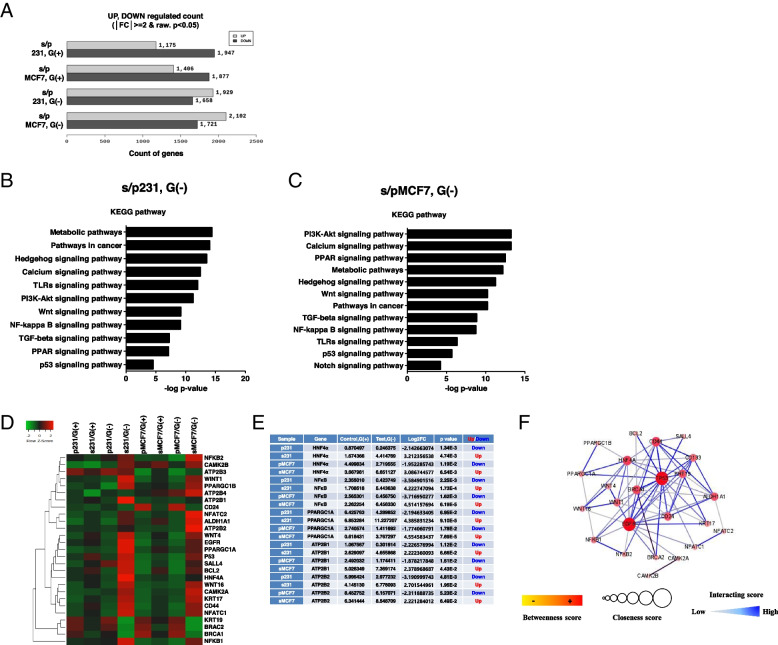
Regulation of target gene expression and signaling pathway in the selected cells under glucose deprivation-induced metabolic stress. **A** Whole gene variance on selected cells under glucose deprivation-induced metabolic stress. The *x*-axis indicates the count of genes in parental and selected cells under glucose-deprived conditions. **B**, **C** Bar plot showing 12 significantly enriched upregulated pathways in selected cells under glucose deprivation-induced metabolic stress. **D** Heatmap of RNA-Seq expression values of target genes in selected cells under glucose deprivation-induced metabolic stress. **E** Significantly enriched upregulated and downregulated calcium regulation-related target genes in selected cells. Control and test were normalized by the trimmed mean of *M*-values (TMM), implemented in the edgeR package. G(+) glucose present, G(−) glucose deprivation, FC fold change. **F** Protein–protein interaction network functional enrichment analysis indicated PGC1α, HNF4α, and NFκB interaction from the STRING database

Previously, we demonstrated that adaptation to chronic metabolic stress led to a positive selection of subclones with CSC characteristics [27]. Furthermore, selected cells showed CSC properties and heightened induction of the survival-signaling pathways compared with their parental lineages under severe ER stress [24]. To identify the changes in gene expression and signaling pathways between glucose deprivation-induced metabolic stress-resistant (selected) and non-resistant (parental) cells, we performed RNA sequencing (RNA-Seq)-based transcriptome analysis. Notably, when subjected to glucose deprivation-induced metabolic stress conditions, selected cells showed induction of target genes and signaling pathways for survival compared with parental cells (Fig. 1A–C). Interestingly, in selected cells, we observed the induction of whole gene expression (Fig. 1A) and activation of gene modules related to mitochondrial pathways, including energy metabolic pathway, calcium, PPAR, and NFκB signaling under glucose deprivation-induced metabolic stress (Fig. 1B, C). Thus, we hypothesized that the survival of selected cells can be attributed to transcriptional activation of gene pathways regulating metabolic, calcium signaling (for overburden of cytosolic free calcium), PPAR signaling (for mitochondrial metabolism), and NFκB signaling pathways (for energy homeostasis and metabolic adaptation) under glucose deprivation-induced metabolic stress. Two well-known transcription factors, HNF4α and NFκB, have direct binding motifs within PGC1α and transcriptional regulators of PMCA [28, 29]. Furthermore, NFκB is a known regulator of energy homeostasis and metabolic adaptation, upregulating mitochondrial respiration [4].

We identified 27 genes as key players in notably different signaling pathways based on Fig. 1A–C. Wnt signaling pathway-related markers of seven genes [30–32], AKT and TGFβ/Smad signaling pathway-related markers of two genes [33–35], p53 signaling pathway-related markers of one gene, calcium signaling pathway-related markers of six genes, PPAR signaling pathway-related markers of two genes, NFκB signaling pathway-related markers of two genes, and apoptosis and stemness-related markers in cancer of seven genes were curated in identified 27 genes. When selected cells were exposed to glucose deprivation-induced metabolic stress (e.g., glucose deprivation), it led to more significant induction of cancer stemness-related genes (e.g., *WNT*, *ALDH1A1*, *EGFR*, and *CD44*), PGC1α, *ATP2B* isoforms, *HNF4α*, and *NFκB* than those in parental cells (Fig. 1D, E) compared with glucose present conditions. PPI network functional enrichment analysis indicated interactions among PGC1α, HNF4α, and NFκB (Fig. 1F). HNF4α and NFκB are known as transcriptional factors of ATP2B1 (PMCA1) and ATP2B2 (PMCA2), respectively (Fig. 1F). Altogether, these data imply that the induction of gene pathways in regulating stemness and transcription factors of PMCAs are key factors for survival under glucose deprivation-induced metabolic stress conditions in selected cells.

### Selected cells resistant to metabolic stress have an increased anti-apoptotic capability by PMCA upon prolonged glucose deprivation

**Fig. 2:**
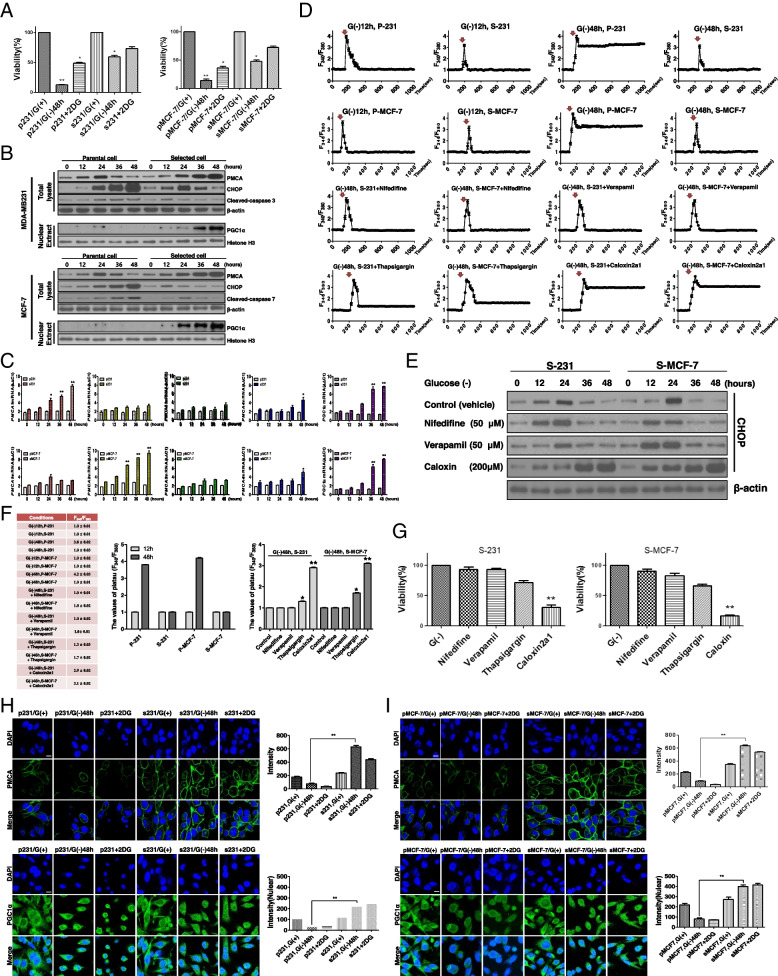
Selected cells survived by PMCA induction to pump calcium to ECM under glucose deprivation-induced metabolic stress. **A** Cell viability assay (MTT) of parental and selected cells under glucose deprivation-induced metabolic stress conditions by 2DG and glucose-deprived conditions. **B** Immunoblot analysis of PMCA, CHOP, PGC1α, caspase-3, and caspase-7 in the parental and selected cells under glucose deprivation-induced metabolic stress conditions at time series. **C** qRT-PCR of parental and selected cells showing the time-dependent mRNA expression of the PMCA family under glucose deprivation-induced metabolic stress conditions. **D** Cytosolic free calcium was measured after treatment with calcium channel blockers (nifedipine or verapamil), SERCA (thapsigargin), or PMCA (caloxin 2a1) inhibitor in parental and selected cells under glucose-deprived conditions in early (12 h) and late (48 h) phases. An arrow indicates the addition of high K^+^ depolarization to induce an increase in Ca^2+^ on traces. **E** Immunoblot analysis of CHOP (ER stress marker) expression after treatment with calcium channel blockers, SERCA, or PMCA inhibitor in selected cells under glucose deprivation-induced metabolic stress conditions at time series. **F** The values of the plateau (*F*_340_/*F*_380_), as the time is 810 s, intracellular Ca^2+^ on MB-231 and MCF-7 cells (left and middle). The values of plateau after treatment with calcium channel blockers (nifedipine or verapamil), SERCA (thapsigargin), or PMCA (caloxin 2a1) inhibitor under glucose-deprived conditions at 48 h in selected cells. **G** Cell viability assay (MTT) of selected cells after treatment of calcium channel blockers and PMCA inhibitors in selected cells under glucose deprivation-induced metabolic stress conditions (48 h). **H**, **I** Immunofluorescence assay for nuclear-translocated PGC1α and PMCA expression in parental or selected cells under glucose deprivation-induced metabolic stress for 48 h. Magnification, × 400. Scale bar, 20 μm. G (−), glucose-deprived condition. Data represent the average of at least three separate independent experiments and presented as means ± SEM. *P < 0.05 vs. parental, **P < 0.01 vs. parental, **P < 0.01 vs. selected G (−), G (+) glucose present, G (−) glucose deprivation

Based on the results of RNA-Seq and Additional file 3: Fig. S2, we hypothesized that PMCA and PGC1α are key regulators of survival under glucose deprivation-induced metabolic stress conditions in selected cells. To evaluate the acquired resistance of the selected cells against glucose deprivation-induced metabolic stress conditions, we performed a cell viability assay. To mimic metabolic stress in the tumor microenvironment over time, selected and parental cancer cells were exposed to glucose deprivation or 2DG (50%) culture media for up to 48 h, generating chronic and gradual metabolic stress. Over time, the number of attached parental cells decreased; however, more than 50% of selected cells remained viable (Fig. 2A). We performed immunoblot analysis to measure target protein expression or nuclear translocation between selected and parental cells (both MDA-MB231 and MCF-7) (Fig. 2B). Both parental cells (P-231 and P-MCF-7) and selected cells (S-231 and S-MCF-7) were subjected to time-dependent (0, 12, 24, 36, 48 h) glucose deprivation. In parental cells, CHOP (ER stress marker) and cleaved caspase 3, 7 (apoptosis markers) levels significantly increased, while PMCA expression and nuclear-translocated PGC1α levels diminished under glucose deprivation. In contrast, in selected cells, PMCA expression and nuclear-translocated PGC1α levels significantly increased, which is associated with a decline in CHOP and cleaved caspase 3, 7 levels (Fig. 2B). PMCA is one of the main free calcium transporters and removes calcium from the cytoplasm. Among the four PMCA isoforms, *PMCA1* and *PMCA2* as well as *PGC1α* mRNA showed the most significant induction in selected 231 and MCF-7 cells, respectively, while no significant induction was observed in parental cells (Fig. 2C). We measured the change of intracellular Ca^2+^ levels using microspectrofluorimetry. Cytoplasmic Ca^2+^ was measured in response to high K^+^ depolarization to induce an increase in cytoplasmic Ca^2+^, and cytoplasmic Ca^2+^ clearance was assessed in the absence and presence of numerous inhibitors of different Ca^2+^ fluxes under glucose-deprived conditions at 12 or 48 h. There were no significant differences in the levels of cytoplasmic Ca^2+^ between parental and selected cells at 12-h glucose deprivation (early phase); however, after 48 h of glucose deprivation (late phase), the levels of cytoplasmic Ca^2+^ were different after the spike of intracellular Ca^2+^. The cytoplasmic Ca^2+^ level of parental cells (P-231 and P-MCF-7) failed to return to the basal levels while that of the selected cells (S-231 and S-MCF-7) returned to the basal levels after the spike of intracellular Ca^2+^ (Fig. 2D, top). These differences in cytoplasmic Ca^2+^ levels between parental and selected cells were correlated with PMCA expression levels. To prove that the main regulator for returned to the basal levels after the spike of intracellular Ca^2+^ after 48 h of glucose deprivation in selected cells was PMCA and not calcium ion channels and SERCA, we treated selected cells with nifedipine or verapamil (both calcium channel blockers), thapsigargin (SERCA inhibitor), and caloxin (PMCA inhibitor) under glucose-deprived conditions (Fig. 2D bottom and Fig. 2E). There were no significant differences in Ca^2+^ flux-treated calcium channel blockers and SERCA inhibitor in selected cells at late phase. Conversely, the treated PMCA inhibitor did not show any differences in the recovery capacity of intracellular Ca^2+^ concentration to basal levels in selected cells (Fig. 2D, bottom). CHOP increased in a time-dependent manner in the caloxin treatment group. However, CHOP decreased in the calcium channel blocker (nifedipine or verapamil) treatment groups (Fig. 2E). When high K^+^ is stimulated, the calcium trace induces peak and plateau on each condition. We observed the values of the plateau (*F*_340_/*F*_380_), as the time is 810 s, intracellular Ca^2+^ on MB-231 and MCF-7 cells. The values of the plateau of P-231 and P-MCF-7 are described to be 3.8 ± 0.2 and 4.2 ± 0.2 after 48 h of glucose-deprived condition (Fig. 2F, left and middle). To verify the mechanism for the change of calcium contents by glucose depletion, it is investigated in various ion channels, SERCA, and PMCA blockers (Fig. 2F, right). As a result, under glucose-deprived conditions at 48 h, cell viability in the nifedipine, verapamil, or thapsigargin treatment groups was not significantly different, while that in the caloxin treatment group was decreased (Fig. 2G). We assessed the expression levels of PGC1α (in the nucleus) and PMCA by immunofluorescence under glucose-deprived conditions in parental and selected cells (both MDA-MB231 and MCF-7) (Fig. 2H, I). PMCA and nuclear-translocated PGC1α expression levels significantly increased in selected cells compared with parental cells under glucose-deprived conditions.

Therefore, these data indicate that selected cells may avoid calcium-mediated apoptosis under glucose deprivation-associated severe ER stress by induction of PMCA.

### Survival of selected cells during prolonged glucose deprivation-induced metabolic stress depends on PGC1α-mediated induction of PMCA expression

**Fig. 3:**
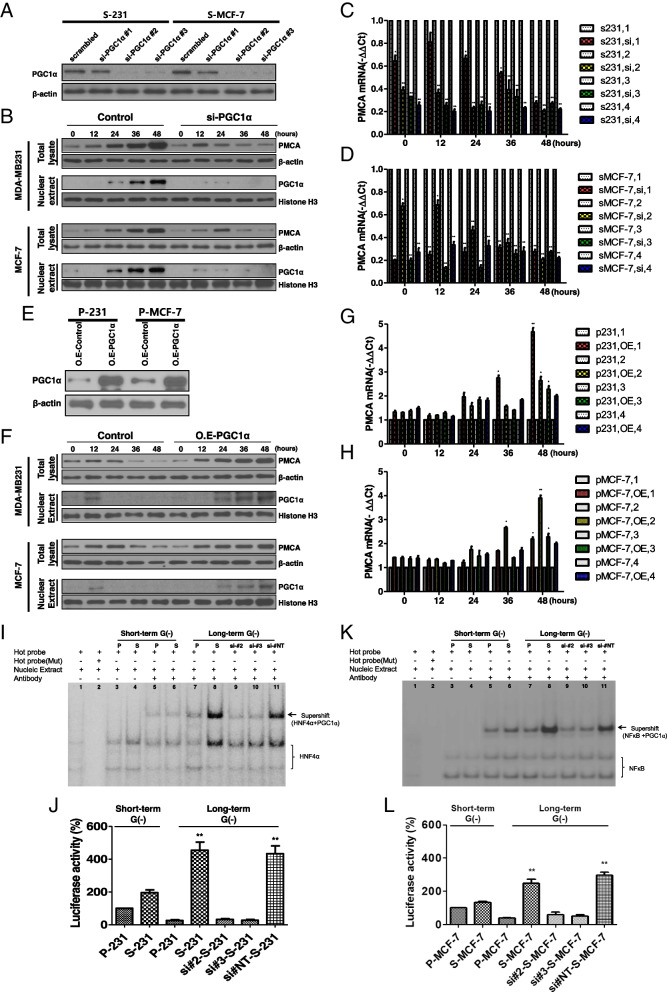
Selected cells survived by induction of PGC1α-mediated PMCA under glucose deprivation-induced metabolic stress conditions. **A**, **B** Immunoblot analysis for PGC1α and PMCA expression in PGC1α knockdown selected cells. Selected cells were transfected with PGC1α siRNA and subjected to glucose deprivation-induced metabolic stress at time series. **C**, **D** qRT-PCR for measurement of mRNA expression of PMCA family under glucose deprivation-induced metabolic stress in PGC1α knockdown selected cells. s231, 1-4 and sMCF-7, 1-4; PMCA1-4. **E**, **F** Immunoblot analysis of PGC1α and PMCA expression in PGC1α-overexpressed parental cells. Parental cells were overexpressed with PGC1α cDNA and subjected to glucose deprivation-induced metabolic stress at time series. p231, 1-4 and pMCF-7, 1-4; PMCA1-4. O.E-PGC1α; PGC1α-overexpressed. **G**, **H** qRT-PCR for measurement of mRNA expression of PMCA family under glucose deprivation-induced metabolic stress in PGC1α-overexpressed parental cells. **I**, **K** EMSA of HNF4α and NFκB, its transcriptional coactivator PGC1α with the PMCA1 and 2 promoters. EMSA was performed with nuclear extract (NE) and a [γ-^32^P]-labeled oligonucleotide, parental and selected cells. **J**, **L** Dual-luciferase reporter assay, which was used to compare HNF4α and NFκB transcriptional activity between parental and selected cells under glucose deprivation-induced metabolic stress in PGC1α knockdown selected cells. Short-term G (−), glucose-deprived conditions for 12 h; long-term G (−), glucose-deprived conditions for 48 h. *P < 0.05 vs. parental or selected, **P < 0.01 vs. selected, **P < 0.01 vs. parental long-term G (−)

To investigate the causal relationship between the induction of PMCA and nuclear-translocated PGC1α, we carried out a knockdown of PGC1α in selected cells (S-231 short interfering [si]-*PGC1α* and S-MCF-7 si-*PGC1α*) (Fig. 3A). Immunoblot analysis (Fig. 3A) showed that the knockdown of *PGC1α* in selected cells using siRNAs S-231 si-*PGC1α* and S-MCF-7 si-*PGC1α* led to reduced levels of PMCA and nuclear-translocated PGC1α under metabolic stress with time-dependent (0, 12, 24, 36, 48 h) glucose deprivation (Fig. 3B). Next, we measured the levels of PMCA isoforms using quantitative real-time RT-PCR. With the knockdown of *PGC1α* in selected cells (S-231 and S-MCF), *PMCA1* (in S-231) or *PMCA2* (in S-MCF-7) reduced as the duration of the glucose deprivation increased in respectively both cell lines (Fig. 3C, D). On the contrary, immunoblot analysis showed that the overexpression of PGC1α in parental cells (P-231 O.E-PGC1α and P-MCF-7 O.E-PGC1α) (Fig. 3E) increased the expression of PMCA protein and nuclear-translocated PGC1α under metabolic stress, with time-dependent (0, 12, 24, 36, 48 h) glucose deprivation (Fig. 3F). With overexpression of PGC1α in parental cells (P-231 and P-MCF-7), *PMCA1* (in P-231) or *PMCA2* (in P-MCF-7) mRNA showed the most significant induction among the four PMCA isoforms (Fig. 3G, H) in respectively both cell lines. These results imply that PGC1α-mediated PMCA induction is a key regulator of survival under glucose-deprived conditions by modulating the cytoplasmic free calcium buffering capacity.

To identify whether PGC1α functions as a transcriptional coactivator by forming a complex with cell type-specific transcription factors HNF4α and NFκB, we investigated the potential HNF4α- and NFκB-binding sites in the PMCA1 and PMCA2 promoter regions. EMSA was performed using a ^32^P-labeled oligonucleotide containing HNF4α- and NFκB-binding sites found in *PMCA1* and *PMCA2* promoters and the HNF4α and NFκB transcription factor complex, including PGC1α, in the presence of a PGC1α antibody (Fig. 3I, K). Nuclear HNF4α and NFκB were more induced in selected cells than parental cells (Additional file 3: Fig. S3). Supershift analysis showed no significant differences between selected and parental cells after short-term (12 h) glucose deprivation [G (−)] (Fig. 3I, K, both lanes 5 and 6), but substantial induction in selected cells under long-term glucose deprivation [G (−)] (Fig. 3I, K, each lane 7 and 8). Upon knockdown of *PGC1α* in selected cells, si-*PGC1α* #2 and si-*PGC1α* #3 showed no significant differences under long-term glucose deprivation (Fig. 3I, K, both lanes 9 and 10). We then examined HNF4α and NFκB transcriptional activation using the HNF4α and NFκB-luciferase reporter assay system, which contained HNF4α- and NFκB-binding sites from the *PMCA1* and *PMCA2* promoters, respectively. A two- to fourfold or higher increase was observed in relative luciferase activity in both S-231 and S-MCF7 cells, particularly after long-term glucose deprivation, than that in their parental cells (Fig. 3J, L, respectively).

Therefore, these results suggest that selected cells may have survived under long-term glucose deprivation by the transport of excess cytoplasmic free calcium to the extracellular space by PMCAs that are transcriptionally activated by PGC1α.

### Survival of stress-resistant CSCs was suppressed by a pharmacophore candidate, functional inhibitor of PMCA

**Fig. 4:**
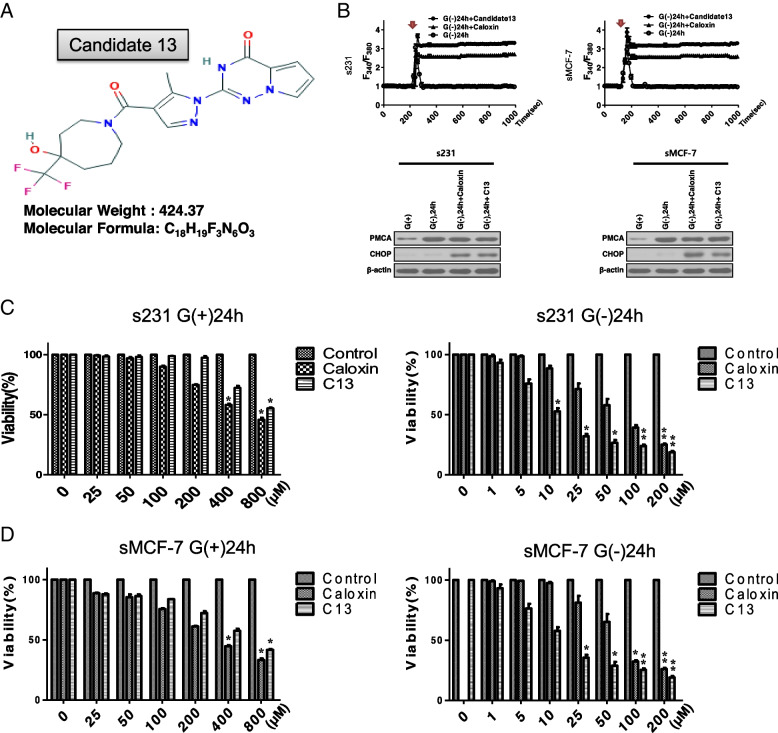
Candidate 13, a novel PMCA inhibitor, in glucose deprivation-induced metabolic stress-resistant cancer cells. **A** Information about candidate 13. **B** Measurement of intracellular free calcium (left) and immunoblot analysis for PMCA expression (right) in selected cells after treatment with PMCA inhibitor, caloxin and candidate 13, under glucose deprivation-induced metabolic stress conditions. An arrow indicates the addition of high K^+^ depolarization to induce an increase in Ca^2+^ on traces. **C**, **D** Viability assay of the selected cells after treatment with caloxin and candidate 13 with or without glucose deprivation-induced metabolic stress conditions. Data represent the average of at least three separate independent experiments. **P < 0.01 vs. G (−), *P < 0.05 vs. control, **P < 0.01 vs. control. G (+) glucose present, G (−) glucose deprivation

Based on the aforementioned results (Figs. 1, 2, and 3), we hypothesized that the inhibition of PMCA could be a potential therapeutic approach for glucose deprivation-induced metabolic stress-resistant cancer cells. In a pharmacophore and docking-based sequential virtual screening for the identification of compounds that bound PMCA, 7201 (docking or scoring), 1028 (manual selection), and 42 candidate compounds were obtained. Among these, 42 compounds bound PMCA with high binding-affinity scores. The 13th candidate (“candidate 13”) showed significant inhibition of PMCA function and was thus selected (Fig. 4A and Additional file 1: Fig. S1). Under glucose-deprived conditions for 24 h, the cytoplasmic Ca^2+^ level was returned to the basal levels after the spike of intracellular Ca^2+^ in selected cells while the treatment of the PMCA inhibitor (caloxin or candidate 13) was failed to return to the basal levels (Fig. 4B, left). PMCA levels (Fig. 4B, right) were increased steadily in selected cells compared to that in the presence of glucose. The change of the PMCA level was not significantly different under glucose-deprived conditions. Nevertheless, treatments with caloxin or candidate 13 showed increased CHOP, a marker of ER stress (Fig. 4B, right). This result showed that caloxin and candidate 13, PMCA inhibitor, could not transcriptionally regulate; it is just a functional inhibitor of PMCA. Consequently, the viability of selected cells treated with caloxin or candidate 13 notably reduced in a dose-dependent manner under glucose-deprived conditions compared to that in the presence of glucose (Fig. 4C, D). In the presence of glucose, even when the dose of caloxin or candidate 13 was increased to 800 μM, the viability of selected cells was maintained (Fig. 4C, D, left panel).

These results imply that PMCA is a key player of survival under glucose-deprived conditions by modulating the elevated cytosolic free calcium buffering capacity.

### A novel therapeutic trial for drug-resistant cancer using PMCA inhibitor candidate 13 in a patient-derived drug-resistant cancer cell mouse xenograft model

**Fig. 5:**
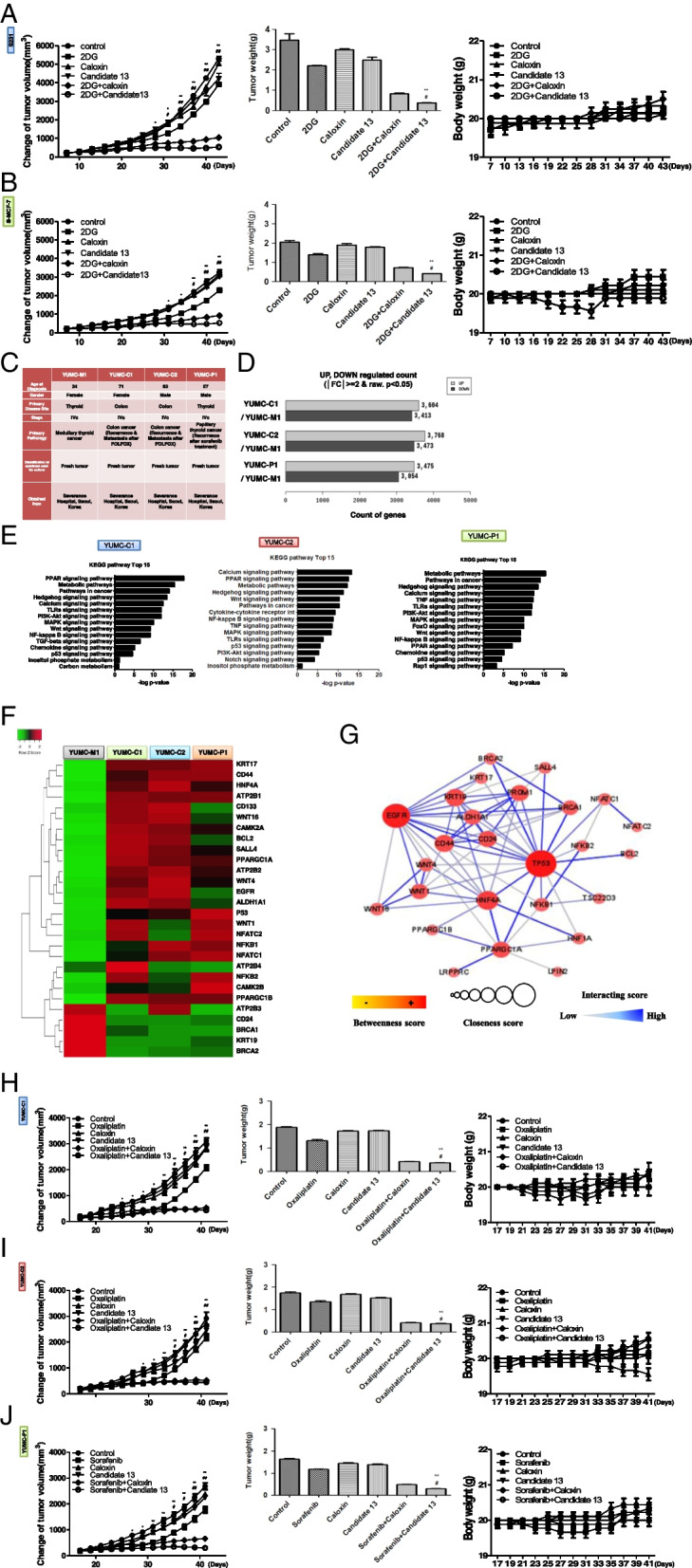
PMCA inhibitor could suppress tumor growth in a xenograft model of metabolic and drug-resistant cancer. **A** (S-231) and **B** (S-MCF-7) Changes in relative tumor volumes (left), dissected tumor weights (middle), and body weight (right) of selected cells (each group, *n* = 10). Tumors were established in athymic nude mice and treated with 2DG (2-deoxy-d-glucose), caloxin, and candidate 13, each agent administered alone or in combination with 2DG. Data are presented as means ± SEM. **C** Characteristics of all examined patient-derived subtypes of cancer cell lines. **D** Whole gene variance in patient-derived drug-resistant cancer cells, YUMC-C1, YUMC-C2, and YUMC-P1, were compared with patient-derived drug-sensitive cancer cells, YUMC-M1. **E** Bar plot showing 15 significantly enriched upregulated pathways in patient-derived drug-resistant cancer cells (top: YUMC-C1, middle: YUMC-C2, bottom: YUMC-P1). **F** Heatmap of RNA-Seq expression values of target genes in patient-derived drug-resistant cancer cells. **G** Protein–protein interaction network functional enrichment analysis indicated PGC1α, HNF4α, and NFκB interaction from the STRING database. **H** (YUMC-C1), **I** (YUMC-C2), and **J** (YUMC-P1) Changes in relative tumor volumes (left), dissected tumor weights (middle), and body weight (right) of patient-derived drug-resistant cancer cells (each group, *n* = 10). Tumors were established in NOG mice and treated with oxaliplatin, sorafenib, caloxin, and candidate 13, each agent treated alone or in combination with oxaliplatin or sorafenib, and caloxin or candidate 13. Data are presented as means ± SEM. *P < 0.05 vs. control, **P < 0.01 vs. control, #*P* < 0.05 vs. oxaliplatin or sorafenib, ##*P* < 0.01 vs. oxaliplatin or sorafenib

Next, we aimed to evaluate the antitumor effects of PMCA inhibitor candidate 13 in vivo*.* We used a mouse xenograft tumor model with selected cancer cells and patient-derived drug-resistant cancer cells. We induced energetic stress in the mouse xenograft model with selected cells by injecting the metabolic inhibitor 2DG, mimicking a glucose deprivation state in vivo. Treatment with 2DG (well-known for its anti-cancer effect), caloxin, or candidate 13 individually showed no significant effect. However, when combined, i.e., 2DG with caloxin or candidate 13, they showed significant induction of tumor shrinkage compared to those in control or individual treatment (Fig. 5A, B, left; Additional file 3: Fig. S4 A and B). The increase in spontaneous ER stress by tumor development and progression caused mild tumor shrinkage with the individual treatment of oxaliplatin, sorafenib, caloxin, and candidate 13 (Additional file 3: Fig. S4 A and B). Furthermore, the excised tumor weight was also significantly lower in the combined treatment group (Fig. 5A, B, middle). Taken together, these results indicate that metabolic stress-resistant selected cells can be killed by selective targeting of PMCA inhibition under glucose deprivation-induced metabolic stress. No evidence of systemic toxicity or treatment-related death was observed in any of the groups. There was also no significant effect on the body weight of mice treated with 2DG, caloxin, or candidate 13, either alone or in combination (Fig. 5A, B, right).

We then tested whether PMCA is a useful therapeutic target in the xenograft mouse model with patient-derived drug-resistant cancer cells, YUMC-C1, YUMC-C2, YUMC-P1 (drug-resistant cancers), and compared with YUMC-M1 (drug-sensitive cancer). Despite clinical therapies using oxaliplatin and sorafenib, these cancer patients experienced therapy failure, cancer recurrence, and metastasis (Fig. 5C). As shown in Fig. 1, we hypothesized that patient-derived drug-resistant cells survive by regulation of target genes and signaling pathways (Fig. 5D, E). We first examined survival-critical genes and signaling pathways in tumor tissues from patients with refractory cancer. As shown in Fig. 5D, E, we assessed a total of 28 key regulator genes in significantly differential signaling pathways in YUMC-C1, YUMC-C2, and YUMC-P1 compared with those in YUMC-M1. The differential mRNA expression patterns between patient-derived drug-resistant and drug-sensitive cancer cells were identified using transcriptome analysis (Fig. 5F). The results of the transcriptome analysis showed that patient-derived drug-resistant cancer cells, when exposed to genotoxic stressors such as oxaliplatin and sorafenib, showed significantly increased levels of cancer stem cell markers (including CD133^high^/CD44^high^/CD24^low^) and expression of survival-related target genes (including PGC1α and ATP2B isoforms) than patient-derived drug-sensitive cancer cells (Fig. 5F). A PPI network functional enrichment analysis indicated interactions among PGC1α, HNF4α, and NFκB (Fig. 5G).

Next, we aimed to identify the antitumor effects of candidate 13 in vivo using patient-derived drug-resistant cancer cells in a mouse xenograft tumor model. Similar to the results shown in Fig. 5A and B, in xenograft models with YUMC-C1, YUMC-C2, and YUMC-P1, treatments with oxaliplatin or sorafenib alone had no significant effect. In contrast, the combined treatments, i.e., oxaliplatin or sorafenib with caloxin or candidate 13, showed significant induction of tumor shrinkage compared with the control or individual treatment group (Fig. 5H–J, left; Additional file 3: Fig. S5A–C). Dissected tumor weights were significantly lower in the combined treatment group (Fig. 5F–H, middle). There was also no significant effect on the body weight of mice treated with oxaliplatin and sorafenib with caloxin or candidate 13 (Fig. 5F–H, right).

Therefore, these data suggest a new therapeutic opportunity to treat drug-resistant cancers, especially those with CSCs.

### CSCs and metabolic- and drug-resistant cancer cell survival rates increased under severe ER stress by induction of PMCA expression

**Fig. 6:**
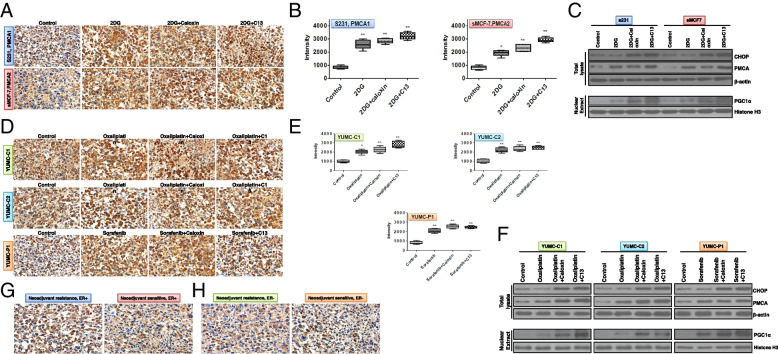
PMCA expression was significantly induced under metabolic or anti-cancer drug stress. Immunohistochemical analysis of PMCA protein levels in paraffin-embedded xenograft tumor tissues with metabolic and drug-resistant (patient-derived) cancer cells. **A** S-231 (top, PMCA1) and S-MCF-7 (bottom, PMCA2) were examined at 400 × magnification; scale bar: 80 μm. **B** S-231 (left) and S-MCF-7 (right), image analysis software was used to quantify the immunostained target proteins. Each assay was performed in triplicate and representative images are displayed. *P < 0.05 and **P < 0.01 vs. control. **C** Immunoblot analysis for CHOP (ER stress marker) expression after treatment of 2DG (2-deoxy-glucose, glucose deprivation-induced metabolic stress inducer) alone or with caloxin or candidate 13 (PMCA inhibitor) in a mouse xenograft model of selected cells (glucose deprivation-induced metabolic stress-resistant cancer cells). **D** Immunohistochemical analysis of PMCA protein levels in paraffin-embedded xenograft tumor tissues with patient-derived drug-resistant cancer cells after treatment of oxaliplatin or sorafenib alone or with caloxin or candidate 13 (PMCA inhibitor). YUMC-C1 (top), YUMC-C2 (middle), and YUMC-P1 (bottom). **E** Each assay was performed in triplicate; representative images were generated with MetaMorph 4.6. YUMC-C1 (left), YUMC-C2 (middle), and YUMC-P1 (right). *P < 0.05 and **P < 0.01 vs. control. **F** Immunoblot analysis for CHOP (ER stress marker) expression after treatment with oxaliplatin or sorafenib alone or with caloxin or candidate 13 in a mouse xenograft model with patient-derived drug-resistant cancer cells. **G**, **H** Immunohistochemical analysis and image analysis of PMCA protein levels in paraffin-embedded patient tumor tissues with neoadjuvant resistance to ER +/− (estrogen receptor positive or negative). **P < 0.01 vs. neoadjuvant resistance/ER+ or ER−. Each assay was performed in triplicate, and representative images are displayed

We showed that the induction of PMCA expression could be a key factor for survival under severe ER stress due to glucose deprivation or anti-cancer drug treatment. Consistent with previous results, immunohistochemistry analysis revealed much higher levels of PMCA in selected cell (S-231 and S-MCF-7)-derived tumor tissues under metabolic stress from 2DG than in control cells (Fig. 6A, B). Notably, selected cells could avoid calcium-mediated apoptosis under metabolic stress via a decrease in ER stress (as indicated by CHOP) by induction of higher PMCA levels (Fig. 6C). Selected cells had induced increased PMCA levels; nevertheless, combined treatments with 2DG and caloxin or candidate 13 showed increased ER stress, induced by caloxin or candidate 13 (Fig. 6C). In line with the results found in selected cells, in patient-derived drug-resistant cancer cells, the expression of PMCA was significantly induced in the oxaliplatin or sorafenib treatment groups compared with the control group (Fig. 6D, E). In contrast, groups that underwent combined treatment with 2DG and caloxin or candidate 13 exhibited increased ER stress (CHOP) due to PMCA inhibition by caloxin or candidate 13 (Fig. 6F). In a neoadjuvant-resistant case of breast cancer, the PMCA level was dramatically higher than that in a neoadjuvant-sensitive case, independent of ER status (estrogen receptor positive/negative, ER+/−) (Fig. 6G, H).

Therefore, these results imply that PGC1α-mediated induction of PMCA is a crucial mechanism by which metabolic stress-resistant cells survived under severe ER stress by nutrient deprivation or anti-cancer drug treatment. In particular, candidate 13 could be a novel therapeutic candidate that may be used to overcome drug-resistant cancer at doses lower than those required when using anti-cancer agents individually.

## Discussion

Drug resistance has continued to be a crucial limiting factor in the successful treatment of cancer patients [36, 37]. Consequently, the identification of therapeutics that can target drug-resistant cancer remains the greatest challenge in cancer research today [38–40]. The fundamental mechanisms of drug resistance in cancer patients vary greatly, because each cancer has its own defining set of features that negate the effect of anti-cancer therapeutics, ultimately instigating cancer progression and death [41, 42]. For this reason, a clinical solution to drug-resistant cancer appears to be an unattainable target. Here, we have proposed the creation of a framework for the therapy of drug-resistant cancer based on the results from representative model experimentation.

The goal of anti-cancer drugs is to achieve the death of cancer cells. However, in our previous study, we discovered that metabolic stress resistance-selected cancer cells avoid calcium-mediated apoptosis under glucose deprivation-induced metabolic stress [24] and phenocopy genotoxic chemotherapy-resistant tumors. Glucose deprivation-induced metabolic stress and anti-cancer drug treatment significantly induces cytosolic free calcium, which leads to the induction of calcium-mediated apoptosis in drug-sensitive cancers [43, 44]. The regulation of cytosolic free calcium signaling is an essential process of resistance to apoptosis by PMCA [20, 45]. PMCA is a ubiquitously expressed protein, essential for maintaining low resting cytosolic free calcium levels in all eukaryotic cells [20]. Moreover, high PMCA expression is associated with poor outcomes in cancer as it protects cells from apoptosis [46]. The glycolytic regulation of PMCA in pancreatic cancer cells has been reported previously [47]. In the study, inhibition of glycolysis induced ATP depletion, thereby inhibiting PMCA, resulting in cytosolic free calcium overload and cell death. Metabolic stressors, such as glucose deprivation, increase cytosolic free calcium by ER stress [12, 48]. The ER is a well-known reservoir of free calcium in the cell; mitochondria also play a role in calcium homeostasis. When cytosolic free calcium levels significantly increase, mitochondria can rapidly uptake cytosolic free calcium for calcium homeostasis by buffering overloaded free calcium. However, sustained cytosolic free calcium overload is deleterious; it eventually decreases mitochondrial respiration, leading to membrane potential decline, mitochondrial swelling, and cytochrome c release and finally triggering apoptotic cell death [49, 50]. Thus, enhanced free calcium exportation through keeping cytosolic free calcium levels low by means of PMCA may explain how selected cells survive sustained glucose deprivation-induced apoptosis.

The present study is the first to show that the transcriptional induction of PMCA via PGC1α plays a critical role in evading calcium-mediated apoptosis under severe ER stress conditions in glucose deprivation-induced metabolic stress-resistant cancer cells. Induction of PMCA by PGC1α contributes to the exporting of overloaded cytosolic free calcium, which is mainly responsible for resistance to such as glucose deprivation-induced ER stress. In addition, increased PMCA expression by PGC1α prevents calcium-dependent apoptosis. PGC1α is well known as a transcription factor that impacts the mass of cellular metabolism [51]. Irregular expression of PGC1α is connected with some chronic diseases and it has been shown to be a crucial modulator of cancer progression [51, 52]. PGC1α functions as a stress sensor in cancer cells and can be stimulated by glucose deprivation, oxidative injury, and chemotherapy. Moreover, PGC1α impacts mitochondrial respiration, ROS (reactive oxygen species) defensive preparations, and fatty acid metabolism through communicating with target-specific transcription factors [52, 53]. In this study, we showed that nuclear translocation of PGC1α could induce target gene expression for survival under prolonged severe ER stress conditions. Of note, this effect was fine-tuned by PGC1α, which transcriptionally co-regulates PMCA expression thereby maintaining cytosolic free calcium homeostasis under glucose-deprived conditions. Furthermore, we propose a clinically translatable approach for significant induction of tumor shrinkage by a novel PMCA inhibitor, candidate 13, in a xenograft tumor model of oxaliplatin- or sorafenib-resistant patient-derived cancer cells. There are numerous transcriptional and signaling pathways responsible for survival in metabolic and chemotherapeutic stress-resistant cancers. Our results only represent part of the entirety of the survival strategy of malignant subtype cancers. Nevertheless, PGC1α-regulated transcriptional activation of PMCA in stress-resistant cancer cells could be potentially exploited as a breakthrough clinical solution to drug-resistant cancer. In conclusion, the present study implies that PGC1α-mediated induction of PMCA is a central mechanism by which metabolic and genotoxic stress-resistant cancer cells survive. Furthermore, we propose a potential clinical approach for significant induction of tumor shrinkage by a novel PMCA inhibitor, candidate compound 13, in a xenograft tumor model of oxaliplatin- or sorafenib-resistant patient-derived cancer cells. Clinically, these observations convey significant implications for the development of novel combinatorial strategies that target the selective vulnerability of highly malignant cells such as drug-resistant and cancer stem-like cells.

## Conclusions

The present study implies that PGC1α-mediated induction of PMCA is a central mechanism by which metabolic and genotoxic stress-resistant cancer cells survive. Furthermore, we propose a clinical approach for significant induction of tumor shrinkage by a novel PMCA inhibitor, candidate compound 13, in a xenograft tumor model of oxaliplatin- or sorafenib-resistant patient-derived cancer cells. Clinically, these observations convey significant implications for the development of novel combinatorial strategies that target the selective vulnerability of highly malignant cells such as drug-resistant and cancer stem-like cells.

## Supplementary Information

**Additional file 1.** Supplementary Methods

**Additional file 2: Suppl. Table 1.** Primer sequences for qRT-PCR. **Suppl. Table 2.** Primer sequences for siPGC1α. **Suppl. Table 3.** Oligomer sequences for EMSA probe.

**Additional file 3: Figure S1.** Scheme of pharmacophore candidates. **Figure S2.** Selected cells survived by induction of PMCA under glucose deprivation induced metabolic stress conditions. Immunoblot analysis for PMCA expression (A and B top), cell viability assay (A and B middle) and intracellular calcium measurements (A and B bottom) after PMCA knockdown in selected cells. A: S-231, B: S-MCF-7. **Figure S3.** Immunoblot analysis of HNF4α and NFκB nuclear translocation. A: P-231 and S-231, B: P-MCF-7 and S-MCF-7. **Figure S4.** Representative images of dissected tumors at completion of treatment schedule with selected cells. A: S-231, B: S-MCF-7. **Figure S5.** Representative images of dissected tumors at completion of treatment schedule with patient-derived cancer cells. A: YUMC-C1, B: YUMC-C2, C: YUMC-P1. **Figure S6.** Changes in relative tumor volumes at different doses of caloxin and candidate 13. A and B: S-231, C and D: S-MCF-7. The dose of 2DG was fixed. Data are presented as mean ± standard error of mean. *P<0.05 versus control (2DG + 100 mg/kg caloxin or 2 DG + 25 mg/kg candidate 13), **P<0.01 versus control (2DG + 100 mg/kg caloxin or 2 DG + 25 mg/kg candidate 13). **Figure S7.** Changes in relative tumor volumes at different doses of oxaliplatin or sorafenib A: YUMC-C1, B: YUMC-C2, C: YUMC-P1. The dose of candidate 13 was fixed. Data are presented as mean ± standard error of mean. *P<0.05 versus control (Candidate 13 + 7.5 mg/kg oxliplatin or candidate 13 + 25 mg/kg sorafenib), **P<0.01 versus control (candidate 13 + 7.5 mg/kg oxliplatin or candidate 13 + 25 mg/kg sorafenib). **Figure S8. ***In vitro* selection process of glucose deprivation induced metabolic stress-resistant selected cells A, Scheme of establishing selected cell sublines. B, Cell growth, glucose consumption and pH changes were traced over time (Top, middle and bottom, respectively). **Figure S9.** A, Immunoblot assay for expression of NCX and PMCA in a dose-dependent manner under glucose-deprived conditions in selected cells. B, Selected cell viability assay treatment with NCX (Bepridil and CKB-R7943), calcium channel (Verapamil and Nifedifine), SERCA (Thapsigargin) and PMCA (Caloxin2a1 and Candidate 13) inhibitors under glucose deprived condition at 48 hours. Points represent the mean percentage of the values determined in the solvent-treated control. * P < 0.05, Caloxin 2a1 vs. Control (selected cells, under glucose deprived conditions at 48 hours), ** P < 0.01, Caloxin 2a1 vs. Control (selected cells, under glucose deprived conditions at 48 hours), * P < 0.05, Candidate 13 vs. Control (selected cells, under glucose deprived conditions at 48 hours), ** P < 0.01, Candidate 13 vs. Control (selected cells, under glucose deprived conditions at 48 hours). Data represent the average of at least three separate independent experiments.

**Additional file 4.**

## Data Availability

All data generated or analyzed during this study are included in this published article and its supplementary information files or from the corresponding author upon reasonable request.

## Abbreviations

2DG: 2-Deoxy-d-glucose
CSCs: Cancer stem cells
EGTA: Ethylene glycol-bis(β-aminoethyl ether)-N,N,N′,N′-tetraacetic acid
EMSA: Electrophoretic mobility shift assay
ER: Endoplasmic reticulum
MTT: 3-(4,5-Dimethylthiazol-2-yl)-2,5-diphenyl tetrazolium bromide
NFκB: Nuclear factor κB
PGC1α: Peroxisome proliferator-activated receptor-γ coactivator 1α
PMCA: Plasma membrane calcium ATPase
PPI: Protein–protein interaction
qRT-PCR: Quantitative reverse transcription PCR
RNA-Seq: RNA sequencing
siRNA: Small interfering RNA
