# Supplementary material for: PMCA inhibition reverses drug resistance in clinically refractory cancer patient-derived models
Source: BMC Med. 2023 Feb 1;21:38. doi: 10.1186/s12916-023-02727-8 (PMC9893610; doi:10.1186/s12916-023-02727-8)
Supplement: Supplementary file 1 — Additional file 1. Supplementary Methods [file 12916_2023_2727_MOESM1_ESM.docx]

**Supplementary Methods**

**Patient characteristics**

**1. Patient 1, YUMC-C1**

Patient 1, YUMC-C1 was a 71-year-old woman with sigmoid colon cancer with liver metastasis who received neoadjuvant folinic acid, oxaliplatin, and fluorouracil (FOLFOX) chemotherapy 7 years prior and underwent anterior resection with wedge resection of the liver. She also underwent four additional wedge liver resections and two intraoperative liver radiofrequency ablations. The specimens for culture were obtained after the last operation. After wedge liver resection (segment 8), the pathology report indicated the presence of metastatic adenocarcinoma.

**2. Patient 2, YUMC-C2**

Patient 2, YUMC-C2 was a 63-year-old man, who was administered neoadjuvant FOLFOX with oxaliplatin and underwent a low anterior resection with wedge liver resection surgery. Two years later, multiple peritoneal masses and a metastatic lesion were found in the liver, and additional surgery was performed. Specimens for culture were obtained from the masses in the liver, and metastatic adenocarcinoma was confirmed in the pathology report.

**3. Patient 3, YUMC-P1**

Patient 3, YUMC-P1 was a 57-year-old man who was recurrent and had metastasis after being administered with sorafenib. After bilateral total thyroidectomy with central compartment neck dissection, this man underwent left radical nephrectomy and right lung wedge resection for kidney and lung metastasis. Afterward, he performed right modified radical neck dissection, left lateral selective lymph node dissection, and 2 regional lymph node dissections. The specimens for culture were obtained after the last operation. After regional lymph node dissection (left level III), the pathology report indicated the presence of metastatic poorly differentiated thyroid carcinoma.

**4. Patient 4, YUMC-M1**

Patient 4, YUMC-M1 was a 24-year-old woman with medullary thyroid carcinoma. She underwent bilateral total thyroidectomy with central compartment neck dissection and bilateral modified radical neck dissection. The specimens for culture were obtained after the first surgery. After surgery, the pathology report indicated the presence of medullary thyroid carcinoma.

**Cell culture**

The patient-derived cancer cells, MDA-MB-231 and MCF-7 cells, were obtained from fresh tumors of patients or ATCC (ATCC, Manassas, VA, USA). YUMC-C1, -C2 and -P1, and -M1 were obtained from colon and papillary thyroid cancer patients treated at the Severance Hospital, Yonsei University College of Medicine, Seoul, Korea. The cells were grown in Roswell Park Memorial Institute-1640 medium, supplemented with 5% (selected cells), 10% (YUMC-M1 and -P1, isolated from patient specimen) and 15% (YUMC-C1 and -C2, isolated from patient specimen) fetal bovine serum. The cells were authenticated using short tandem repeat profiling, karyotyping, and isoenzyme analysis. Mycoplasmal contamination was checked for with the Lookout Mycoplasma PCR Detection Kit (Sigma-Aldrich, St. Louis, MO, USA; MP0035). Further protocol details are as described in our previous article [1, 2]

**In vitro selection process of metabolic stress-resistant selected cells**

Briefly, we hypothesized that adaptation to chronic metabolic stress would positively select subclones with enhanced survival competence. In order to mimic metabolic stress in the tumor microenvironment over time, we maintained cancer cells in culture without changing or replenishing culture media for up to 30 days generating chronic and gradual metabolic stress (Supplementary Figure 8). Scheme of establishing selected cell sublines (Supplementary Figure 8A). Change of surviving P-231 and MCF-7 cells according to changes in glucose and pH(Supplementary Figure 8B). Over time the number of attached cells decreased; however, in each case a small number of viable cells were present in each. At the end of the selection process defined by no changes in cell number over 3 consecutive 2 day-interval measurements, we replenished the cultures with fresh medium with 5% FBS and established ‘selected cell’ sublines.

**mRNA-Seq data**

We preprocessed the raw reads from the sequencer to remove low quality and adapter sequences before analysis and aligned the processed reads to the *Homo sapiens* genome assembly (GRCh37) using HISAT v2.1.0 (KIM et al, 2015). HISAT utilizes two types of indexes for alignment: a global, whole-genome index, and tens of thousands of small local indexes. Both are constructed using the same Burrows–Wheeler transform (BWT) or graph FM index (GFM) as Bowtie2. Because of the use of these efficient data structures and algorithms, HISAT generates spliced alignments several times faster than Bowtie and the widely used BWA. The reference genome sequence of Homo sapiens (GRCh37) and annotation data were downloaded from the National Center for Biotechnology Information (NCBI). Then, transcript assembly of known transcripts was processed using StringTie v2.1.3b (Pertea, Mihaela, et al., 2015, 2016). Based on these results, expression abundance of transcript and gene were calculated as read count or fragments per kilobase of exon per million fragments mapped (FPKM) value per sample. The expression profiles were used for additional analyses, such as of differentially expressed genes (DEGs). In groups with different conditions, differentially expressed genes or transcripts were filtered through statistical hypothesis testing.

**Protein–protein interaction (PPI) analysis through the STRING database**

Protein-protein interaction(PPI) was analyzed based on the STRING database and visualized using Cytoscape. PPI contains physical and functional associations. The STRING database contains various sources of information (KEGG, GO) and involving experimental data, computationally predicted interaction from text mining (pubmed, SGD) and computed from genomic features. PPI was sorted by combined score (combined score ≥ 0.4 was considered the threshold value) and connecting total genes counts (>3 counts). The combined score is computed by combining the probabilities from the different evidence channels and corrected for the probability of randomly observing an interaction. Cytoscape, open-source software platform, was visualized complex networks and that converted to figure form.

**Statistical analysis of gene expression level**

The relative abundances of genes were measured in Read Count using StringTie. We performed statistical analyses to find differentially expressed genes using the estimates of abundances for each gene in the samples. Genes with one more than zeroed Read Count values in the samples were excluded. To facilitate log2 transformation, 1 was added to each Read Count value of filtered genes. Filtered data were log2-transformed and subjected to trimmed mean of M-values (TMM) normalization. The statistical significance of the differential expression data was determined using exactTest, edgeR and fold change, in which the null hypothesis was that no difference exists among groups. False discovery rate (FDR) was controlled by adjusting the p-value using the Benjamini-Hochberg algorithm. For DEG sets, hierarchical clustering analysis was performed using complete linkage and Euclidean distance as a measure of similarity. Gene-enrichment and functional annotation analysis and pathway analysis for significant gene list were performed based on Gene Ontology and KEGG pathway analyses.

**Cell lines, stable transfection of siPGC1α, and overexpression of PGC1α**

The CSC MDA-MB231 and MCF-7 cells were transfected with PGC1α siRNA or control scrambled siRNA, according to the manufacturer's protocol. The sequence of the PGC1α siRNA was designed using siDESIGN (Bioneer, Daejun, Korea); the siRNA duplex was purchased from Bioneer. Further details are as described in our previously published article [3]. For overexpression of PGC1α, expressing PGC1α was used and empty cmv10-flag vector was used as the control vector. PGC1α overexpression was confirmed by immunoblot analysis using anti-PGC-1α (Abcam, #54481).

**Down regulated PMCA in the selected cells using PMCA siRNA**

The selected MDA-MB231 and MCF-7 cells were transfected with PMCA siRNA or control scrambled siRNA, according to the manufacturer's protocol. The sequence of the PMCA siRNA was predesigned using AccuTarget™ (Genome-wide predesigned siRNA, Bioneer, Daejun, Korea, PMCA1; #490-1, PMCA2; #491-1, Control; #SN-1003); the siRNA duplex was purchased from Bioneer.

**Electrophoretic mobility shift assay (EMSA)**

The DNA binding activity of HNF4α and NFκB against the PMCA1 and 2 promoters was confirmed using a ^32^P-labeled oligonucleotide. specific labeled and unlabeled oligonucleotides are as follows supplementary table 2. Nuclear extracts were prepared and isolated using NE-PER Nuclear and Cytoplasmic Extraction Reagents (Pierce, Rockford, IL, USA; 78833) and protein extraction solution (PRO-PREP, iNtRON Biotechnology, Seoul, Korea, 17081). The DNA binding activity of hepatocyte nuclear factor 4 α (HNF4α) and nuclear factor (NF) κB against PMCA1 and 2 promoters was confirmed using a ^32^P-labeled oligonucleotide. Nuclear extracts-DNA interactions were carried out in a 30 μL reaction mixture containing 5 μg of nuclear protein extract, 50 mM HEPES (pH 7.8), 300 mM KCl, 1% Igepal, 30% glycerol, 1 mM DTT, 0.02 μg of poly (dI-dC), 0.1 μg of ssDNA, and 20 000 CPM of ^32^P-radiolabeled oligonucleotide. For the supershift assay, 5 μg of anti-PGC1α antibodies (Abcam, Cambridge, UK, #106814) were added to the mixture of nuclear extracts-DNA and incubated for one additional hour at 4 °C. Further protocol details are as described in our previous article [2].

**Dual luciferase assays**

Promoter activity was evaluated using the Dual-Luciferase Reporter Assay (Promega, Madison, WI, USA; E1960), according to the manufacturer's protocol. Regions of HNF4α and NFκB binding sites were amplified by PCR from human genomic DNA. Sequences are HNF4α: Forward 5′-ATCTTGACCTTTGGCCCATGA-3, Reverse 5′-TCATGGGCCAAAGGTCAAGAT-3′, NFκB: Forward 5ʹ-GGGGGGTTCCC-3ʹ, Reverse 5′-GGGAACCCCCC-3′, siPGC1α #2: Forward 5′-GGACAGUGAUUUCAGUAAUTT-3′, Reverse 5′-AUUACUGAAAUCACUGUCCTT-3′, siPGC1α #3: Forward 5′-CACCACUCCUCCUCAUAAATT-3′, Reverse 5′-UUUAUGAGGAGGAG UGG UGTT-3′). The PCR products were cloned into the pGL4.70 promoter Vector (Promega) using T4 DNA ligase (Thermo Scientific, Waltham, MA, USA; EL0011). All insertions were confirmed by sequencing. Cells were co-transfected with a plasmid containing the 3XκB-Luc reporter, as well as with a Renilla luciferase in pGL4.70 (as the control). Luciferase detection was carried out 48 h after reporter transfection. Expression was estimated as the relative Firefly luciferase activity normalized to the activity of transfection control Renilla luciferase.

**Immunohistochemistry**

Immunohistochemical staining was performed using a standard protocol. Tumor tissues were fixed in 10% formaldehyde and were embedded in paraffin. Tissue sections (5 μm) were dewaxed, and antigen retrieval was carried out in citrate buffer (pH 6), with an electric pressure cooker set at 120 °C for 8 min. Sections were incubated for 10 min in 3% hydrogen peroxide to quench endogenous tissue peroxidase. Primary antibodies against PMCA1 (1:25, Abcam, Cambridge, UK, #3528), PMCA2 (1:25, Abcam, #3529), and PMCA-pan (1:50, Abcam, #2825) were diluted with phosphate-buffered saline. All primary antibody incubation was perfomed overnight at 4°C. Especially process of blocking and antibody incubation for PMCA2 IHC in this study, when the blocking process was short (RT, less than an hour), a lot of backgroud was observed. We recommend procees of blocking and primary antibody incubation each overnight at 4°C for PMCA2 IHC. All tissue sections were counterstained with hematoxylin, dehydrated, and mounted.

**In vivo mouse xenograft study**

Cancer cells obtained from ATCC (MDA-MB-231 and MCF-7; 1.0 × 10^6^ cells/mouse) and cancer cells isolated from patients (YUMC-C1, -C2 and -P1 cells; 4.5 × 10^6^ cells/mouse) were cultured *in vitro*, then injected subcutaneously into the upper left flank region of 6-week-old female BALB/c nude and NOD/Shi-scid, IL-2Rγ KOJic (NOG) mice. Tumor size was measured using calipers; tumor volume was then estimated using the following formula: L × S^2^/2 (where L and S are the longest and shortest diameters, respectively). After 7 - 15 d, when the tumor sizes reached volumes of approximately 100–200 mm^3^, tumor-bearing mice were randomly grouped (n = 7-10/group) to receive 17 mg/kg oxaliplatin alone (p.o.), 80 mg/kg sorafenib alone (p.o.), or a combination of 7.5 mg/kg oxaliplatin (p.o.) and 25 mg/kg sorafenib (p.o.) once every 3 d. 500 mg/kg 2DG (p.o.), 100 mg/kg caloxin 2a1 (Plasma Membrane Calcium ATPase inhibitor) (i.v.), 25 mg/kg candidate 13 (p.o.) treatments were given equal dosage either alone or in combination. Dose of 2DG was based on the previous article [4]. Caloxin, candidate 13, oxaliplatin and sorafenib doses were selected by pilot experiments with different doses (Supplementary figure 6A-D or 7A-C). Animals were maintained under specific pathogen-free conditions, and all experiments were approved by the Animal Experiment Committee of Yonsei University.

**References**

1. Kim SM, Kim SY, Park CS, Chang HS, Park KC: **Impact of Age-Related Genetic Differences on the Therapeutic Outcome of Papillary Thyroid Cancer.** *Cancers (Basel).* 2020,**12**(2).

2. Park KC, Kim SW, Jeon JY, Jo AR, Choi HJ, Kim J, et al: **Survival of Cancer Stem-Like Cells Under Metabolic Stress via CaMK2alpha-mediated Upregulation of Sarco/Endoplasmic Reticulum Calcium ATPase Expression.** *Clin Cancer Res.* 2018;**24**(7):1677-90.

3. Yang H, Yang R, Liu H, Ren Z, Wang C, Li D, et al: **Knockdown of peroxisome proliferator-activated receptor gamma coactivator-1 alpha increased apoptosis of human endometrial cancer HEC-1A cells.** *Onco Targets Ther*. 2016;**9**:5329-38.

4. Cheong JH, Park ES, Liang J, Dennison JB, Tsavachidou D, Nguyen-Charles C, et al: **Dual inhibition of tumor energy pathway by 2-deoxyglucose and metformin is effective against a broad spectrum of preclinical cancer models**. *Mol Cancer Ther*. 2011;**10**(12):2350-62.
