# Supplementary material for: PMCA inhibition reverses drug resistance in clinically refractory cancer patient-derived models
Source: BMC Med. 2023 Feb 1;21:38. doi: 10.1186/s12916-023-02727-8 (PMC9893610; doi:10.1186/s12916-023-02727-8)
Supplement: Supplementary file 2 — Additional file 2: Suppl. Table 1. Primer sequences for qRT-PCR. Suppl. Table 2. Primer sequences for siPGC1α. Suppl. Table 3. Oligomer sequences for EMSA probe. [file 12916_2023_2727_MOESM2_ESM.docx]

**Supplementary Tables**

Suppl. Table 1. Primer sequences for qRT-PCR.

| Gene products | Forward primer | Backward primer |
| --- | --- | --- |
| *PMCA1* | TTTCCAAACACTGCTTCTCTTC | GGTCCACAGATGCATTACGA |
| *PMCA2* | GTTTTAGGCACTTTTGTGGT | CTAATTCCTCCTCAGGTATT |
| *PMCA3* | AGGCCTGGCAGACAACACCA | TCCCACACCAGCTGCAGGAA |
| *PMCA4* | GAGCTTCCTGGATACCGATG | CTAGCTTGGCCACACTG |
| *PGC1α* | TGAGAGGGCCAAGCAAAG | ATAAATCACACGGCGCTCTT |
| *α-tubulin* | CGGGCAGTGTTTGTAGACTTGG | CTCCTTGCCAATGGTGTAGTGC |

Suppl. Table 2. Primer sequences for siPGC1α.

| Gene products | Forward | Reverse |
| --- | --- | --- |
| *siRNA-1* | 5′-GUCGCAGUCACAACACUUATT-3′ | 5′-UAAGUGUUGUGACUGCGACGCGACTT-3′ |
| *siRNA-2* | 5′-GGACAGUGAUUUCAGUAAUTT-3′ | 5′-AUUACUGAAAUCACUGUCCTT-3′ |
| *siRNA-3* | 5′-CACCACUCCUCCUCAUAAATT-3′ | 5′-UUUAUGAGGAGGAG UGG UGTT-3′ |
| *Scrambled* | 5′-UUCUCCGAACGUGUCACGUTT-3′ | 5′-ACGUGACACGUUCGGAGAA TT-3′ |

Suppl. Table 3. Oligomer sequences for EMSA probe.

| Gene products | TFs | Forward | Reverse |
| --- | --- | --- | --- |
| HNF4α | PMCA1 | 5′-ATCTTGACCTTTGGCCCATGA-3′ | 5′-TCATGGGCCAAAGGTCAAGAT-3′ |
| mut-HNF4α | PMCA1 | 5′-ATCaTcACCTTTGGCCCATGA-3′ | 5′-TCATGGGCCAAAcGaCAAGAT-3′ |
| NFκB | PMCA2 | 5ʹ-GGGGGGTTCCC-3ʹ | 5′-GGGAACCCCCC-3′ |
| mut-NFκB | PMCA2 | 5ʹ-GGGcGcTTCCC-3ʹ | 5′-GGGAAgCgCCC-3′ |
