# Supplementary material for: PMCA inhibition reverses drug resistance in clinically refractory cancer patient-derived models
Source: BMC Med. 2023 Feb 1;21:38. doi: 10.1186/s12916-023-02727-8 (PMC9893610; doi:10.1186/s12916-023-02727-8)
Supplement: Supplementary file 3 — Additional file 3: Figure S1. Scheme of pharmacophore candidates. Figure S2. Selected cells survived by induction of PMCA under glucose deprivation induced metabolic stress conditions. Immunoblot analysis for PMCA expression (A and B top), cell viability assay (A and B middle) and intracellular calcium measurements (A and B bottom) after PMCA knockdown in selected cells. A: S-231, B: S-MCF-7. Figure S3. Immunoblot analysis of HNF4α and NFκB nuclear translocation. A: P-231 and S-231, B: P-MCF-7 and S-MCF-7. Figure S4. Representative images of dissected tumors at completion of treatment schedule with selected cells. A: S-231, B: S-MCF-7. Figure S5. Representative images of dissected tumors at completion of treatment schedule with patient-derived cancer cells. A: YUMC-C1, B: YUMC-C2, C: YUMC-P1. Figure S6. Changes in relative tumor volumes at different doses of caloxin and candidate 13. A and B: S-231, C and D: S-MCF-7. The dose of 2DG was fixed. Data are presented as mean ± standard error of mean. *P<0.05 versus control (2DG + 100 mg/kg caloxin or 2 DG + 25 mg/kg candidate 13), **P<0.01 versus control (2DG + 100 mg/kg caloxin or 2 DG + 25 mg/kg candidate 13). Figure S7. Changes in relative tumor volumes at different doses of oxaliplatin or sorafenib A: YUMC-C1, B: YUMC-C2, C: YUMC-P1. The dose of candidate 13 was fixed. Data are presented as mean ± standard error of mean. *P<0.05 versus control (Candidate 13 + 7.5 mg/kg oxliplatin or candidate 13 + 25 mg/kg sorafenib), **P<0.01 versus control (candidate 13 + 7.5 mg/kg oxliplatin or candidate 13 + 25 mg/kg sorafenib). Figure S8. In vitro selection process of glucose deprivation induced metabolic stress-resistant selected cells A, Scheme of establishing selected cell sublines. B, Cell growth, glucose consumption and pH changes were traced over time (Top, middle and bottom, respectively). Figure S9. A, Immunoblot assay for expression of NCX and PMCA in a dose-dependent manner under glucose-deprived conditions in selected [file 12916_2023_2727_MOESM3_ESM.pptx]

## Slide 1
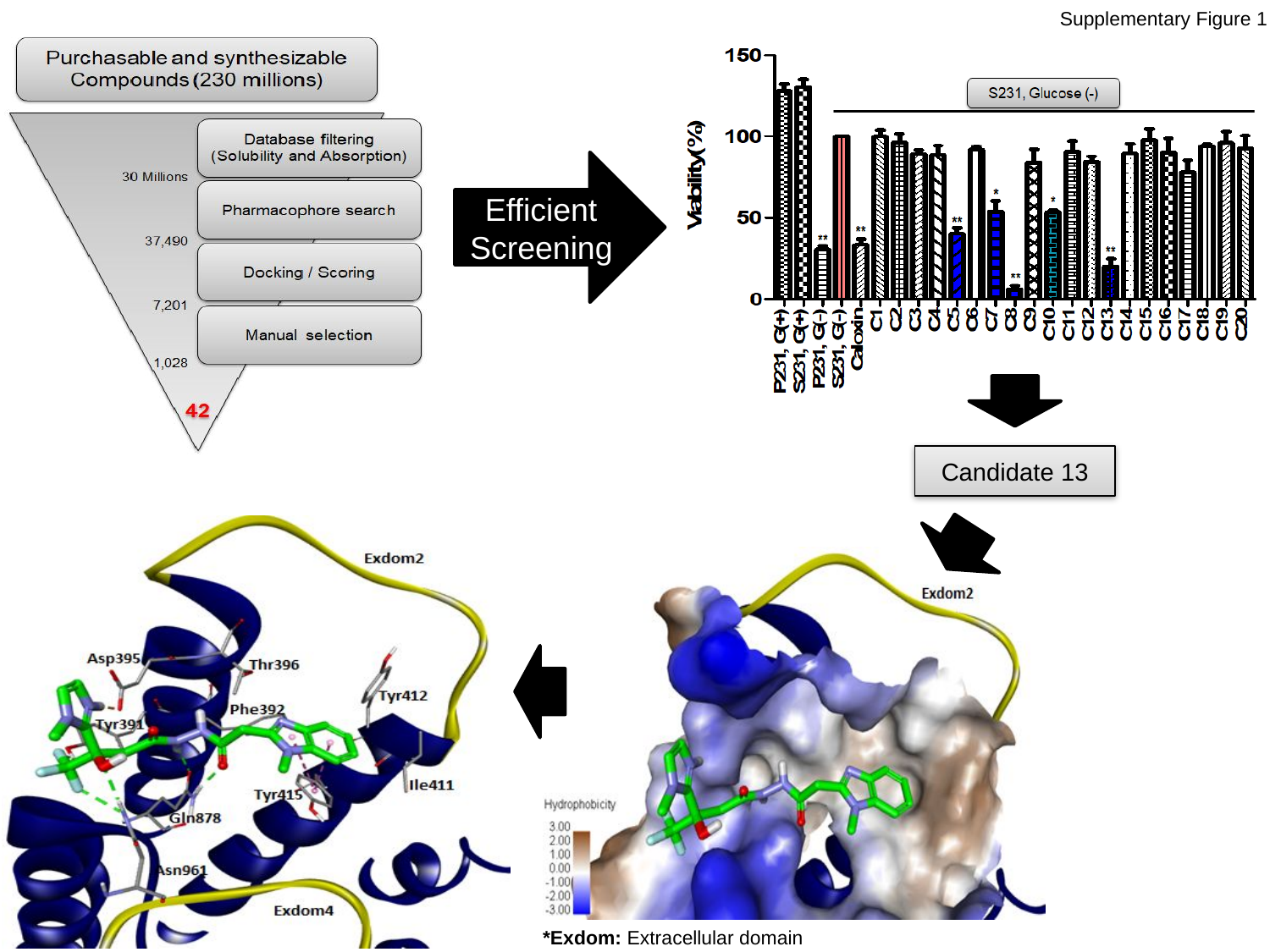

Supplementary Figure 1
Efficient Screening
Candidate 13
*Exdom: Extracellular domain

## Slide 2
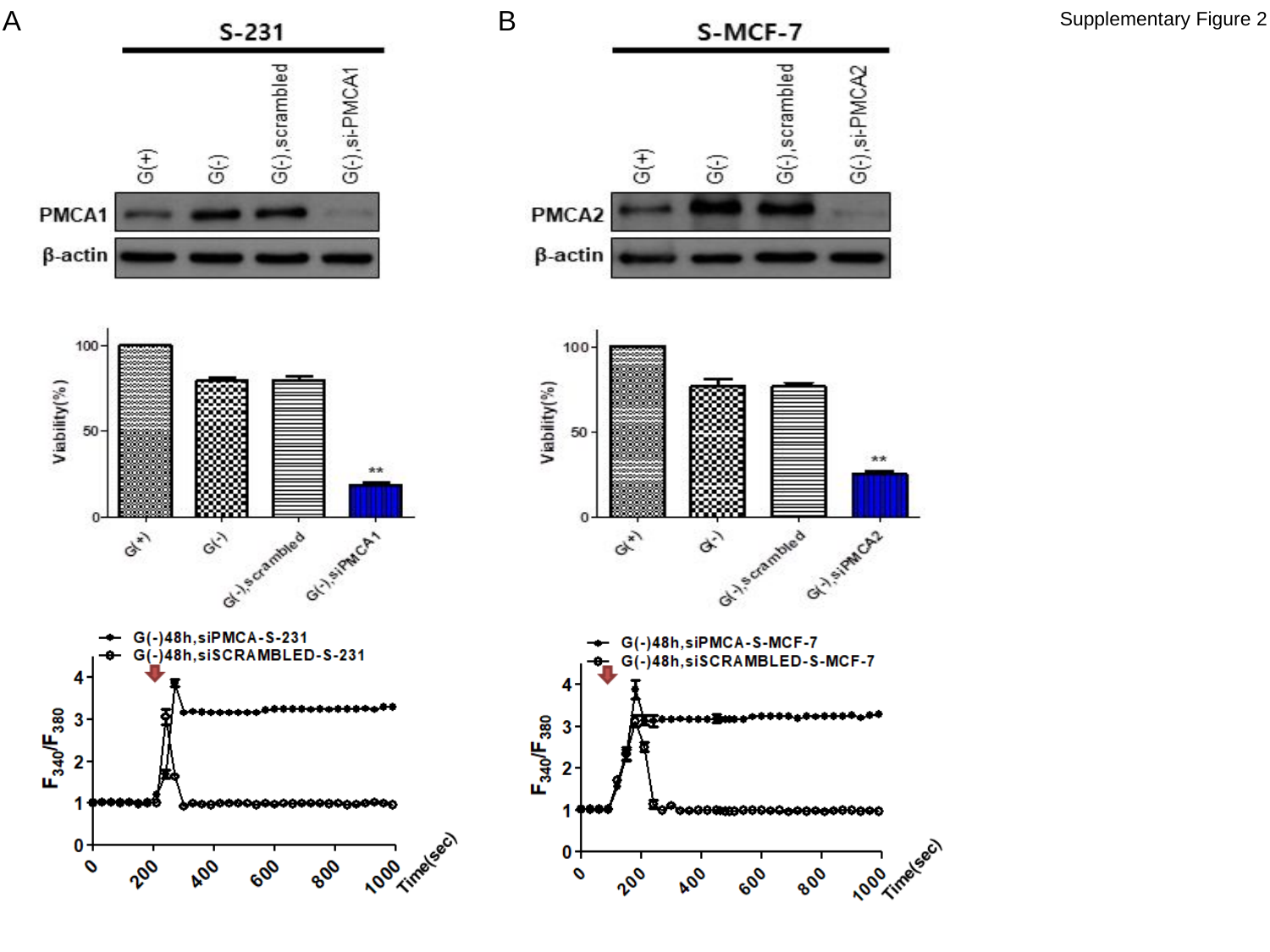

A
B
Supplementary Figure 2

## Slide 3
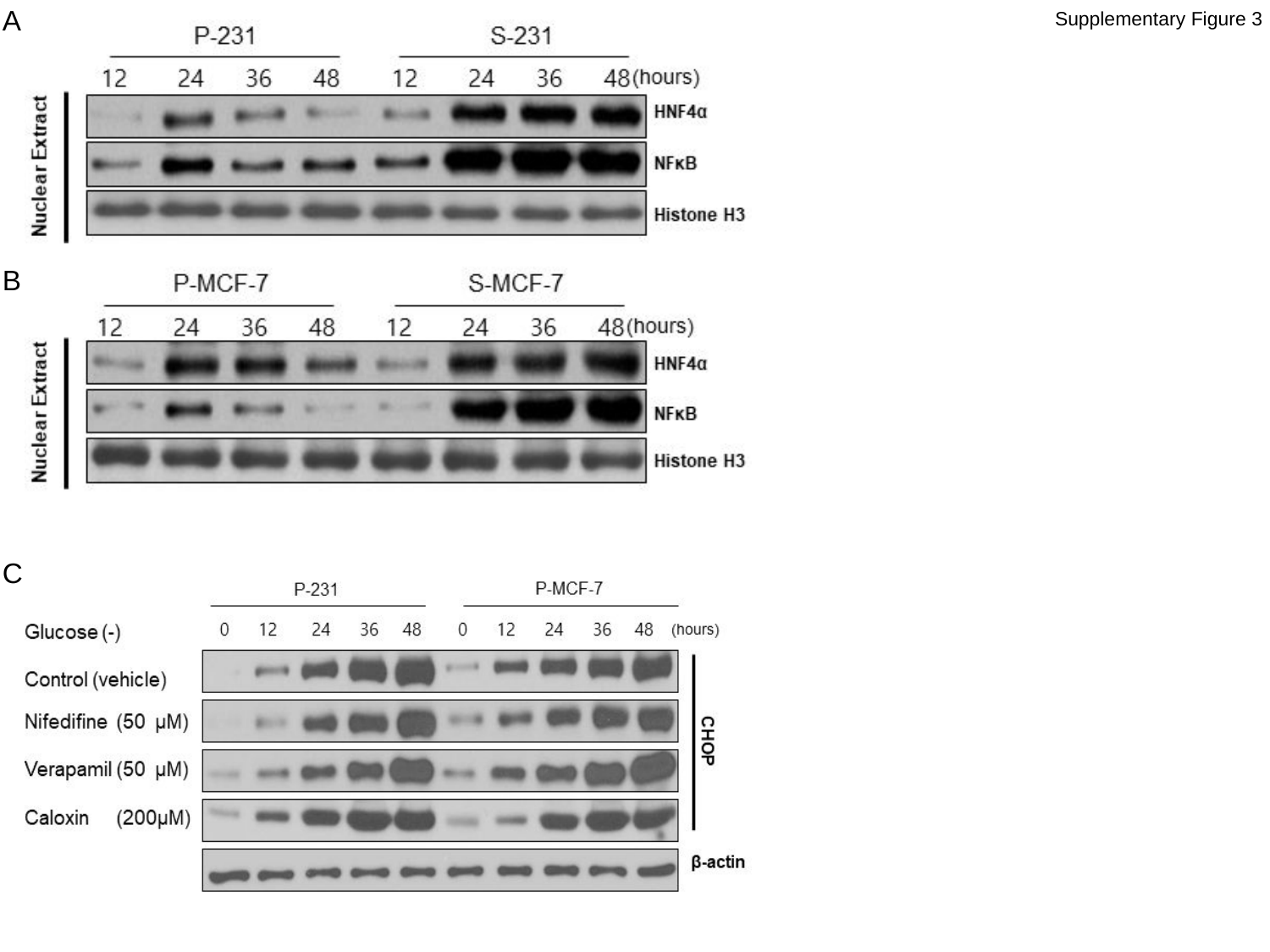

Supplementary Figure 3
A
B
C

## Slide 4
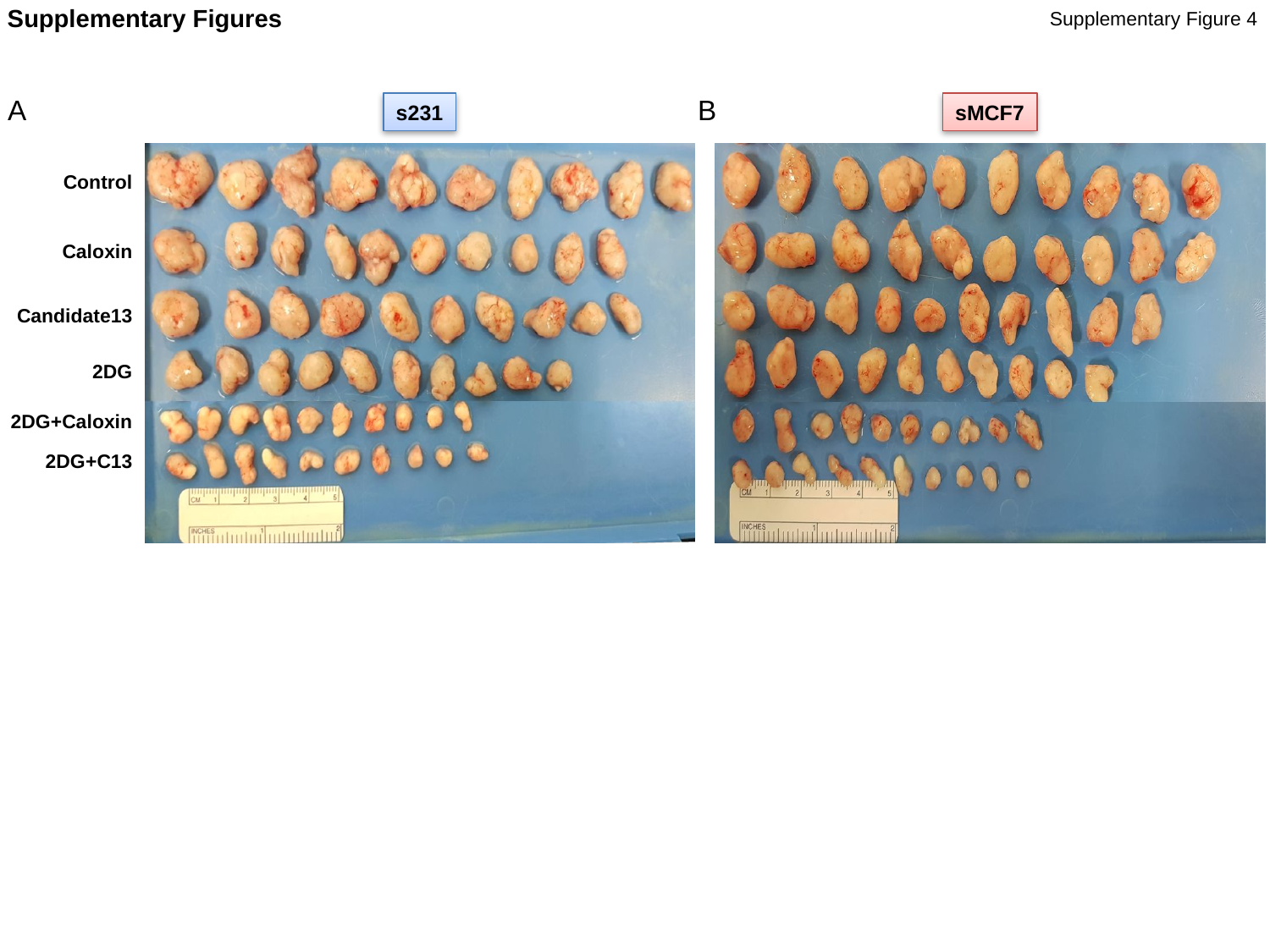

Supplementary Figures
Supplementary Figure 4
A
B
s231
sMCF7
Control
Caloxin
Candidate13
2DG
2DG+Caloxin
2DG+C13

## Slide 5
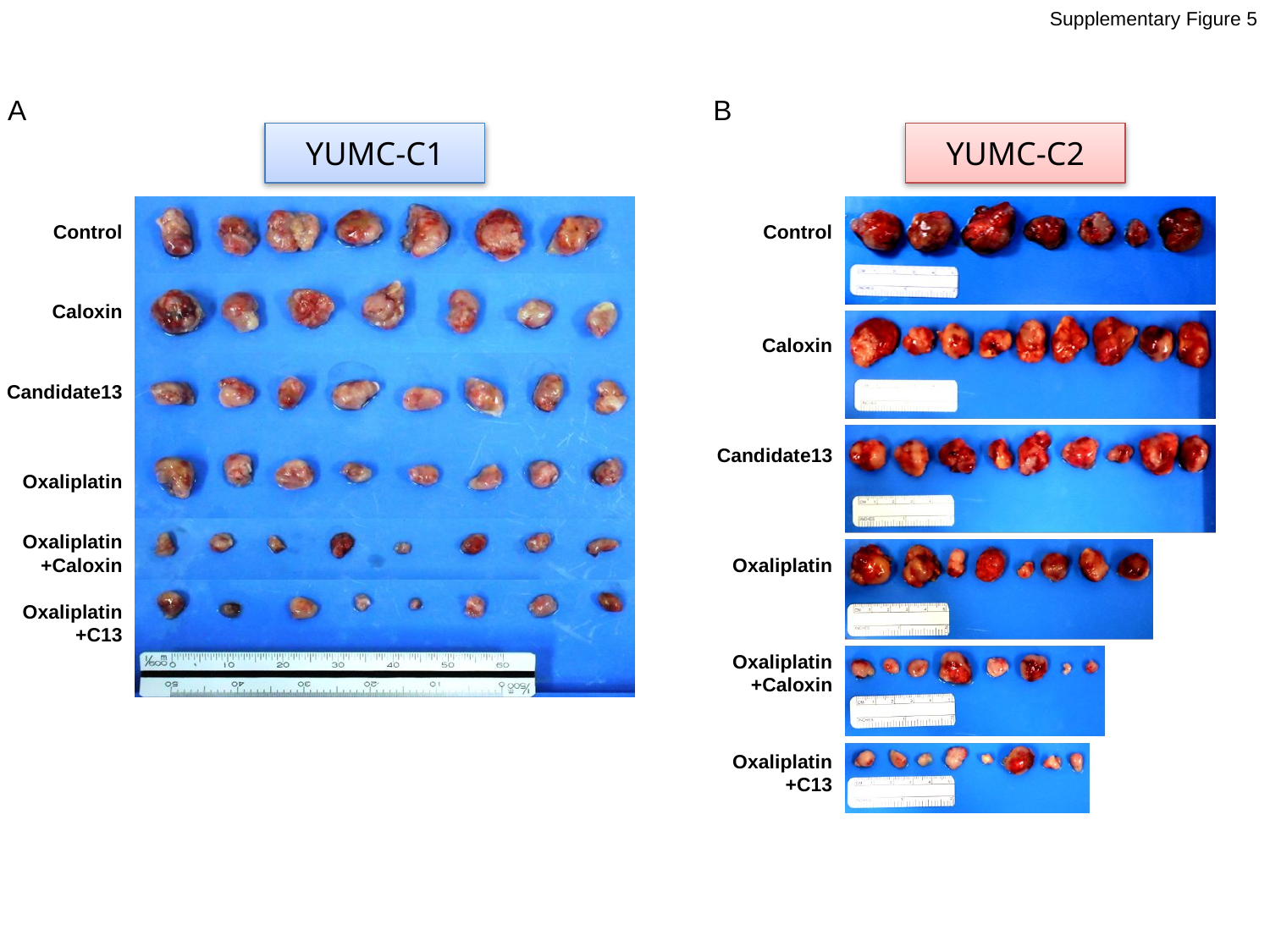

Supplementary Figure 5
A
B
YUMC-C1
YUMC-C2
Control
Control
Caloxin
Caloxin
Candidate13
Candidate13
Oxaliplatin
Oxaliplatin
+Caloxin
Oxaliplatin
Oxaliplatin
+C13
Oxaliplatin
+Caloxin
Oxaliplatin
+C13

## Slide 6
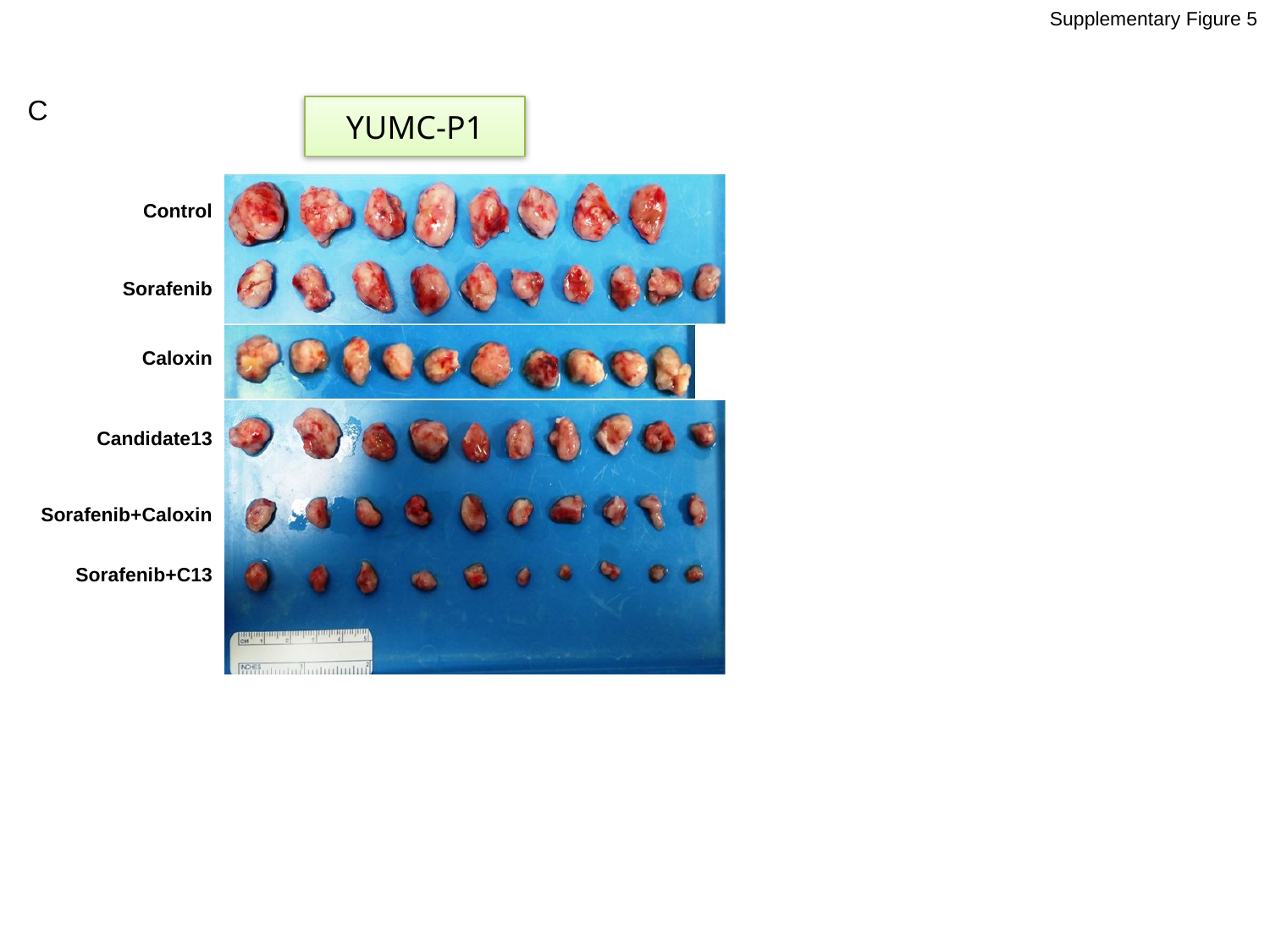

Supplementary Figure 5
C
YUMC-P1
Control
Sorafenib
Caloxin
Candidate13
Sorafenib+Caloxin
Sorafenib+C13

## Slide 7
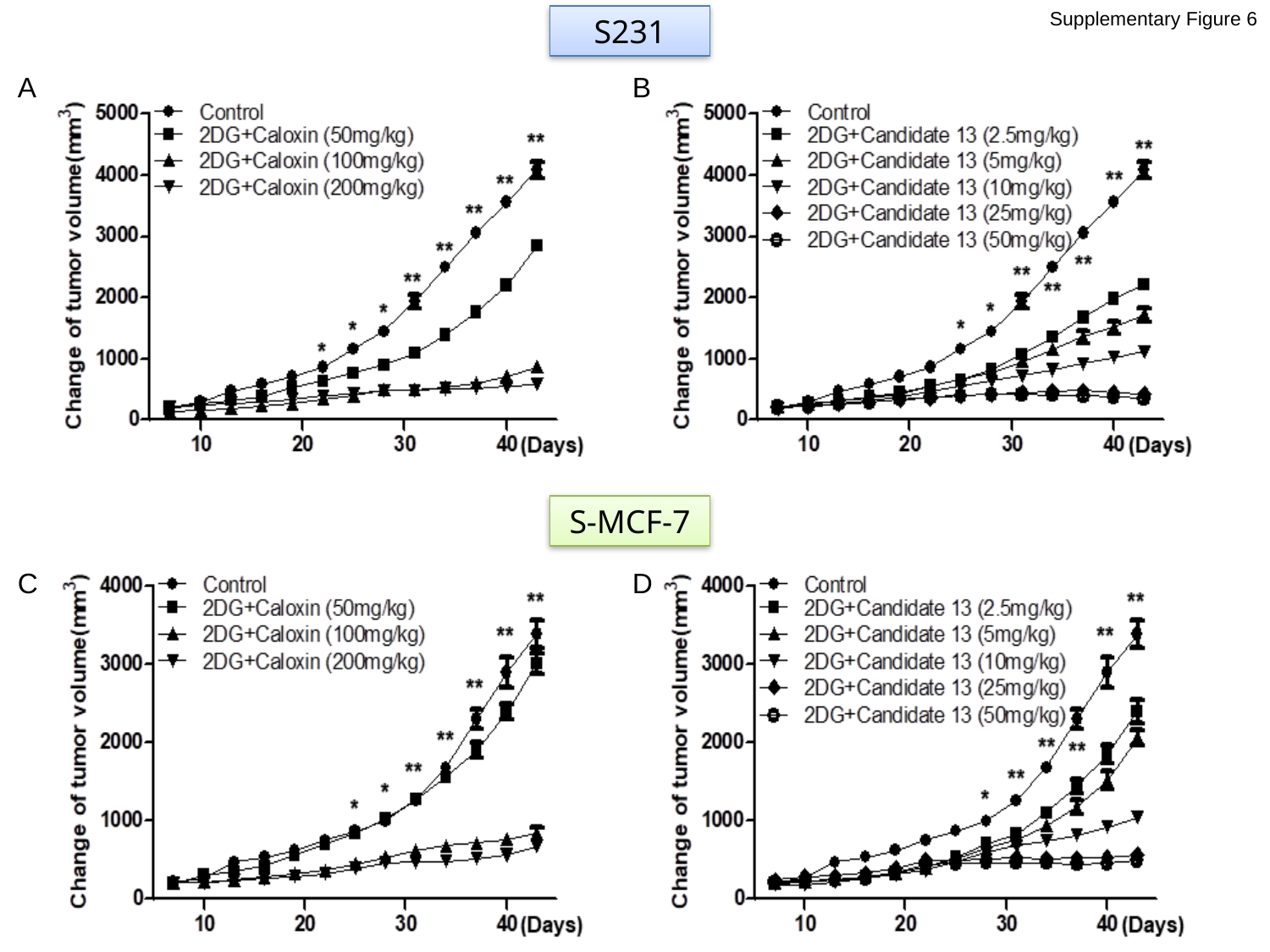

Supplementary Figure 6
S231
A
B
S-MCF-7
C
D

## Slide 8
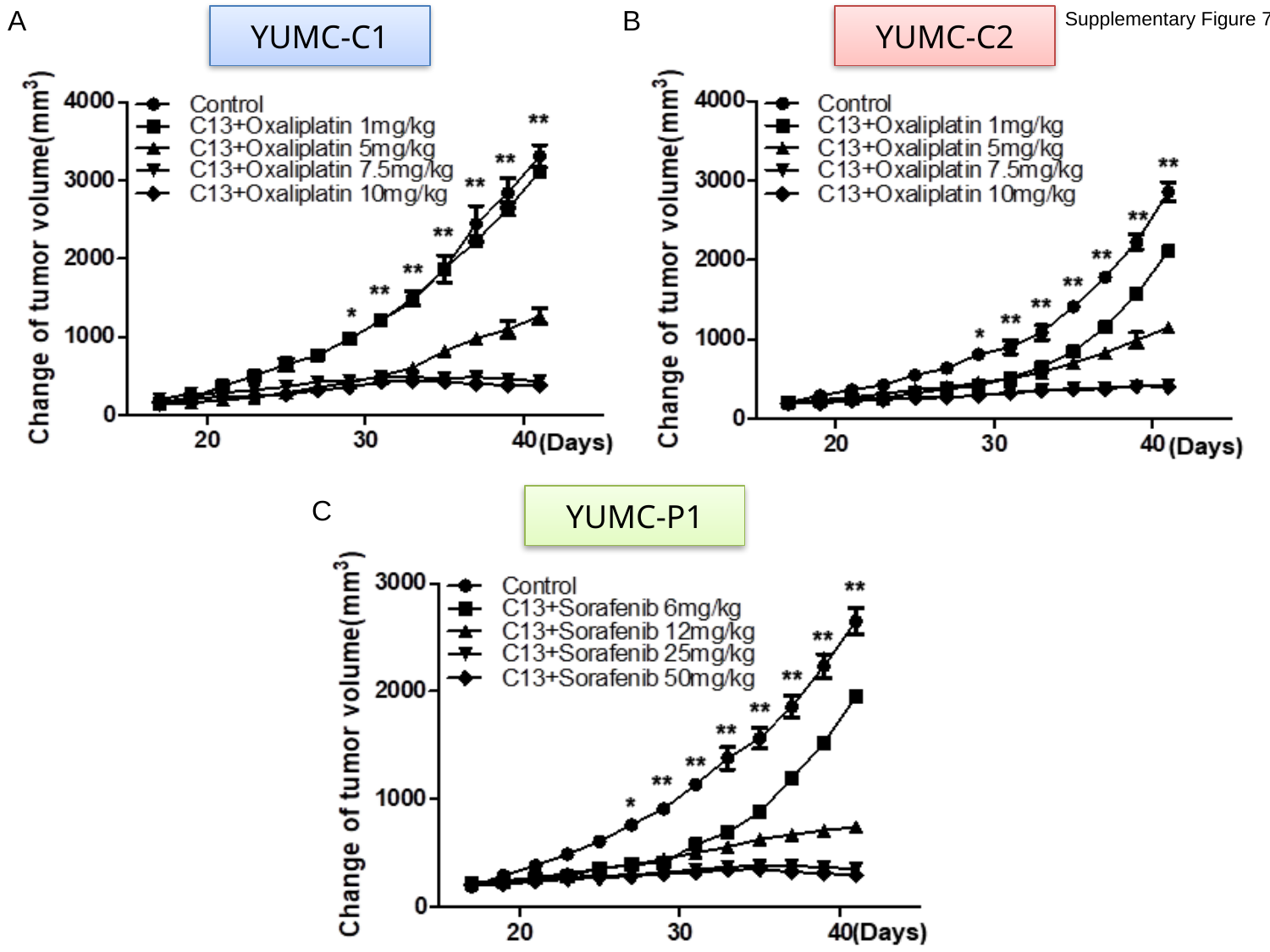

Supplementary Figure 7
A
B
YUMC-C1
YUMC-C2
C
YUMC-P1

## Slide 9
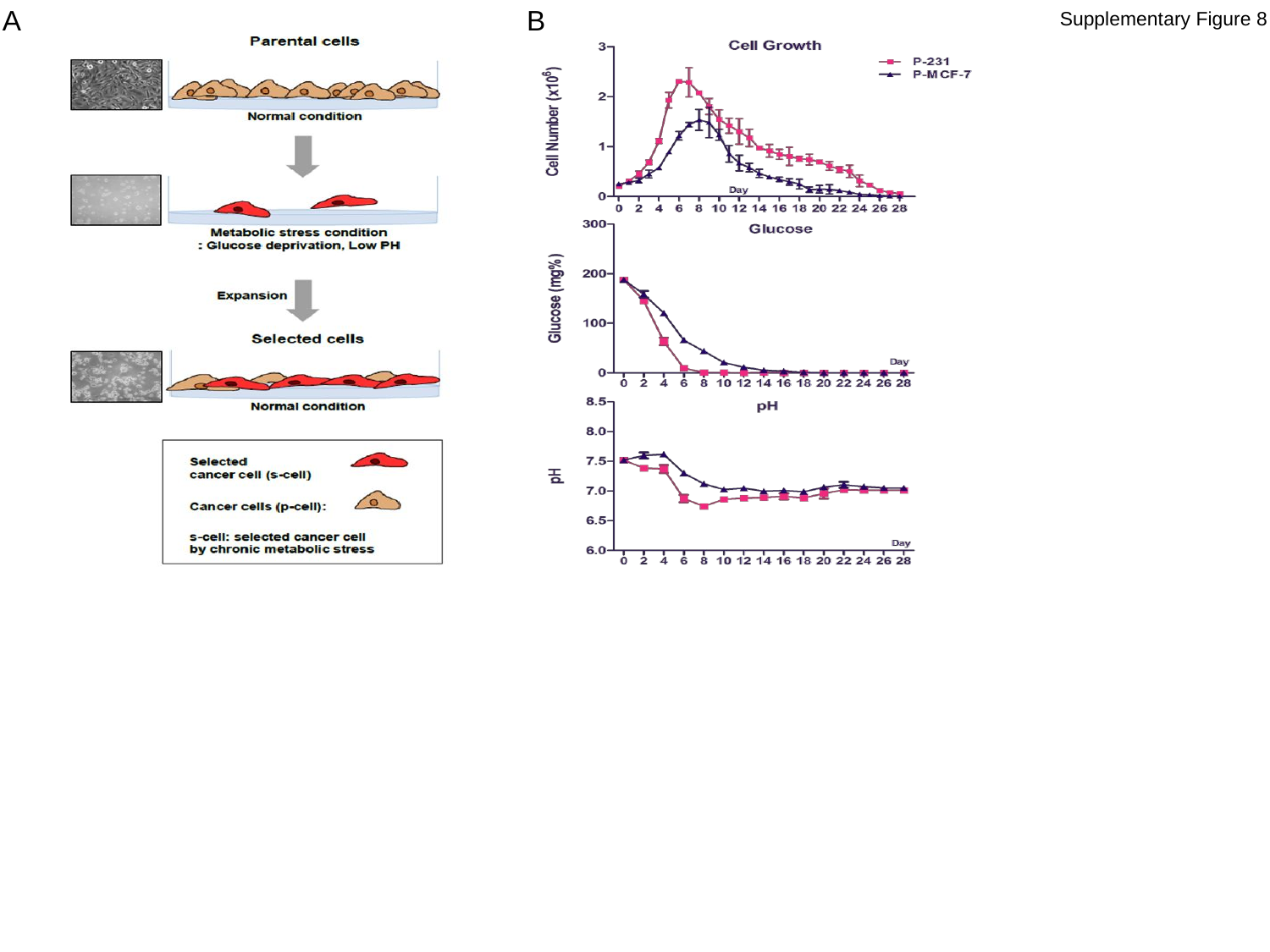

Supplementary Figure 8
A
B

## Slide 10
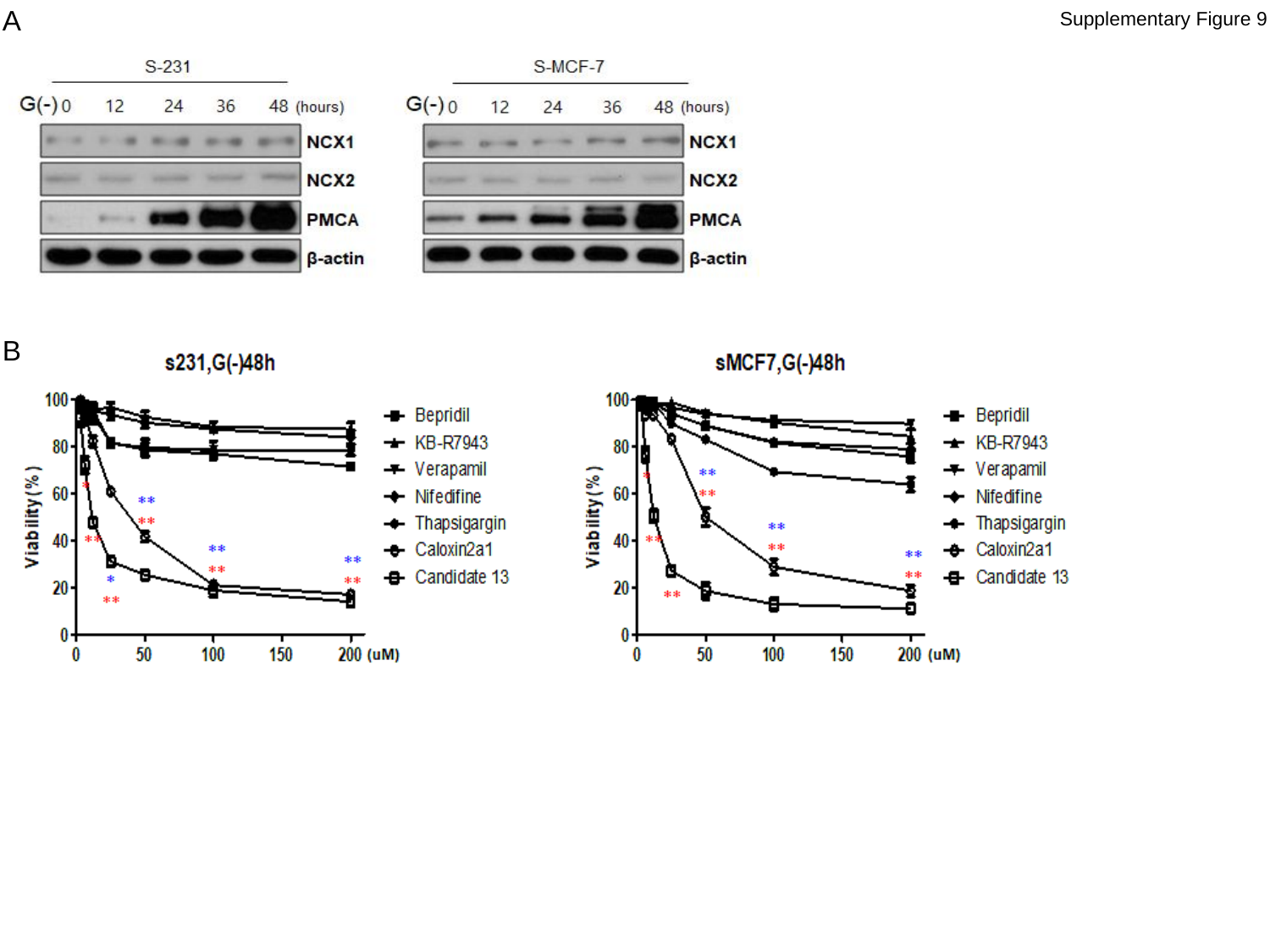

Supplementary Figure 9
A
B
