# Supplementary material for: PMCA inhibition reverses drug resistance in clinically refractory cancer patient-derived models
Source: BMC Med. 2023 Feb 1;21:38. doi: 10.1186/s12916-023-02727-8 (PMC9893610; doi:10.1186/s12916-023-02727-8)
Supplement: Supplementary file 4 — Additional file 4. [file 12916_2023_2727_MOESM4_ESM.pptx]

## Slide 1
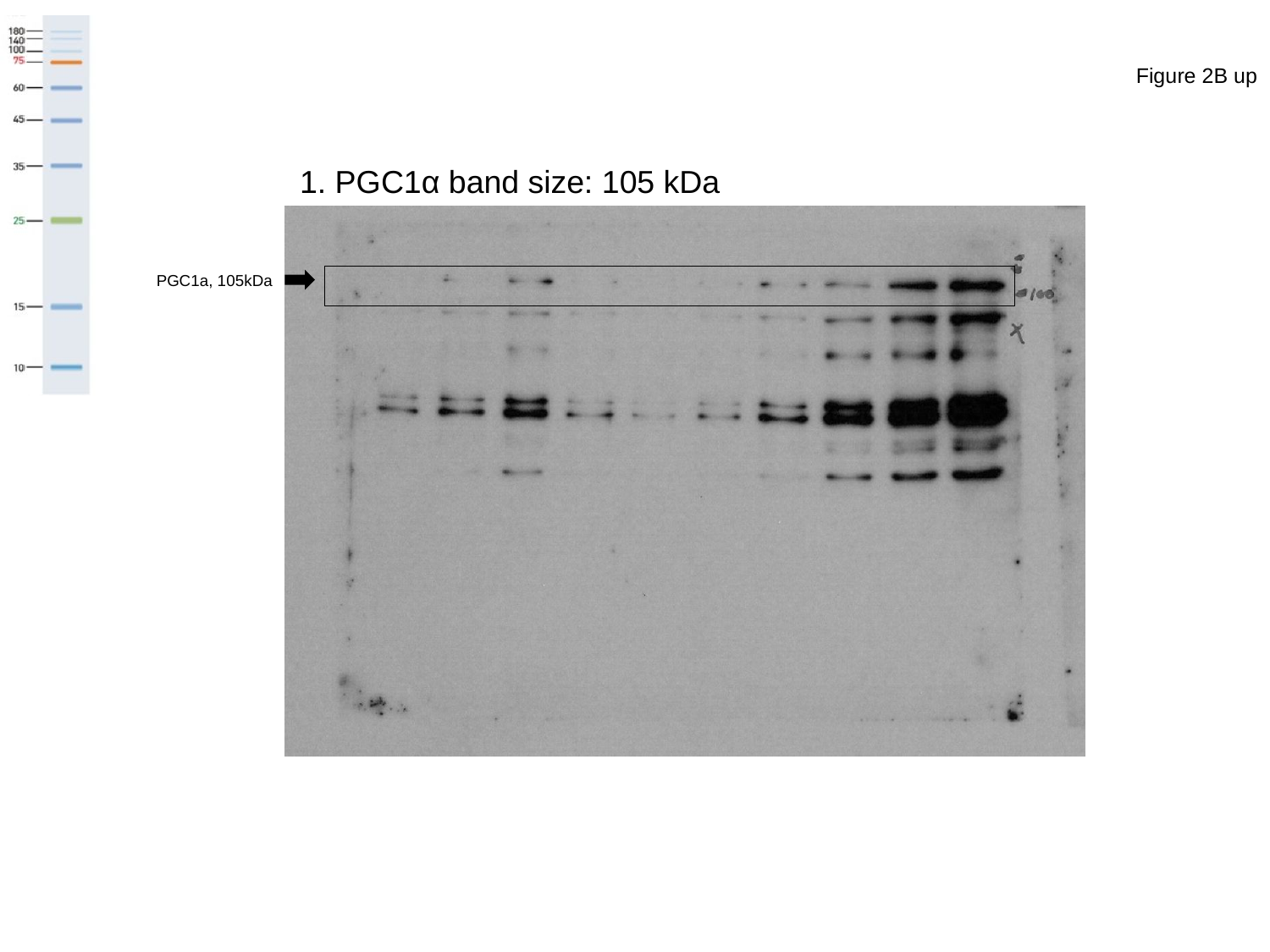

Figure 2B up
1. PGC1α band size: 105 kDa
PGC1a, 105kDa

## Slide 2
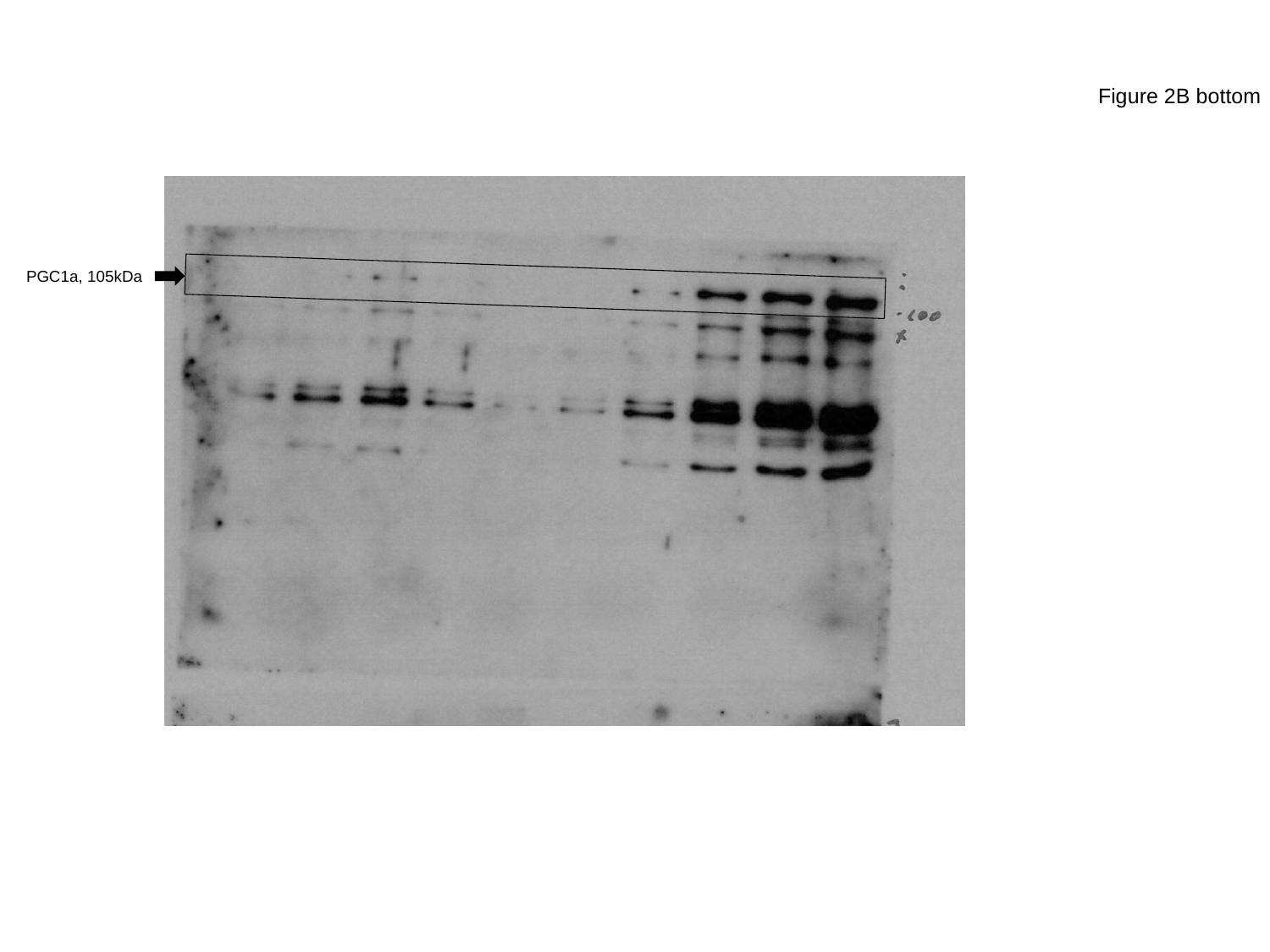

Figure 2B bottom
PGC1a, 105kDa

## Slide 3
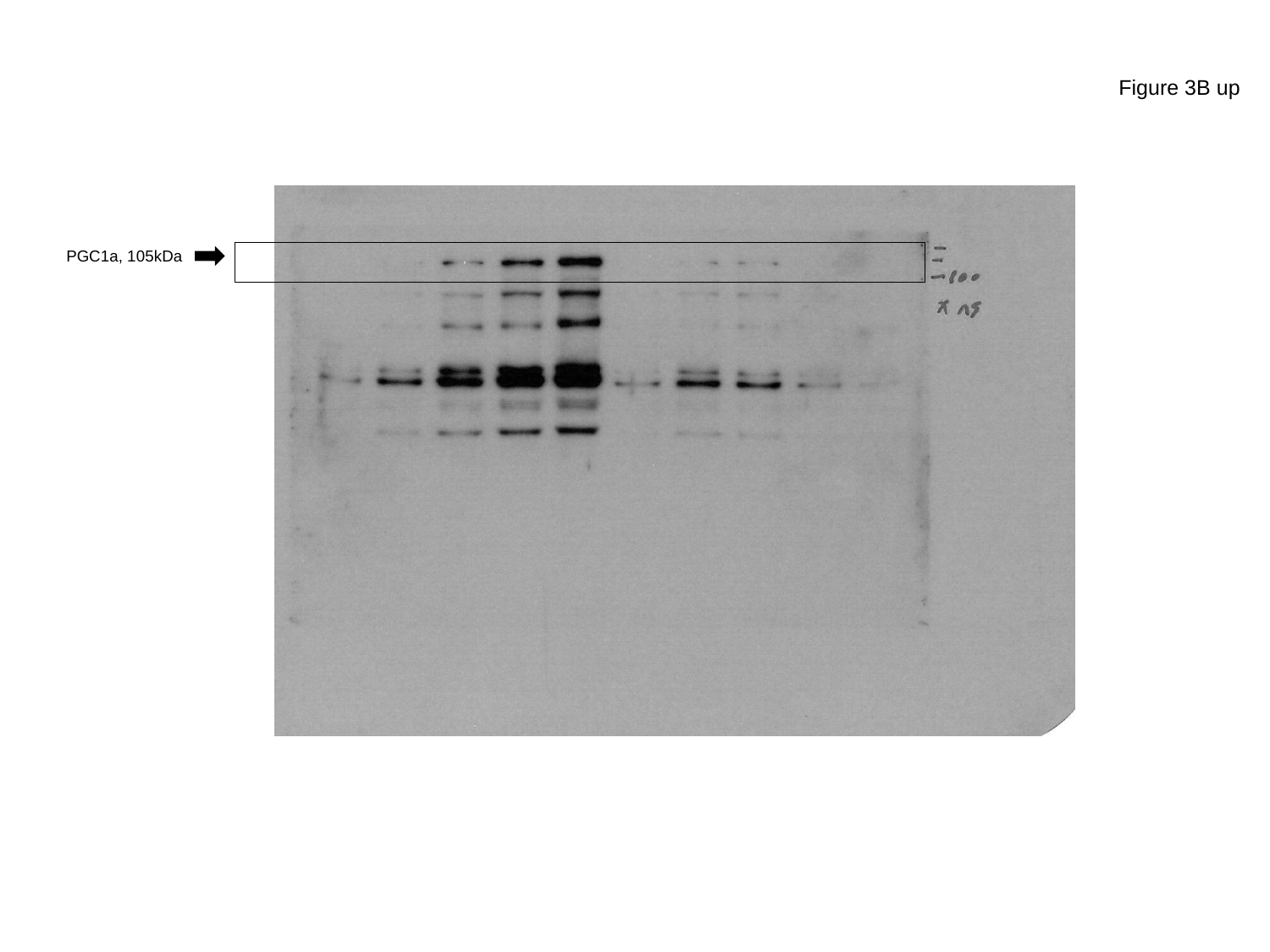

Figure 3B up
PGC1a, 105kDa

## Slide 4
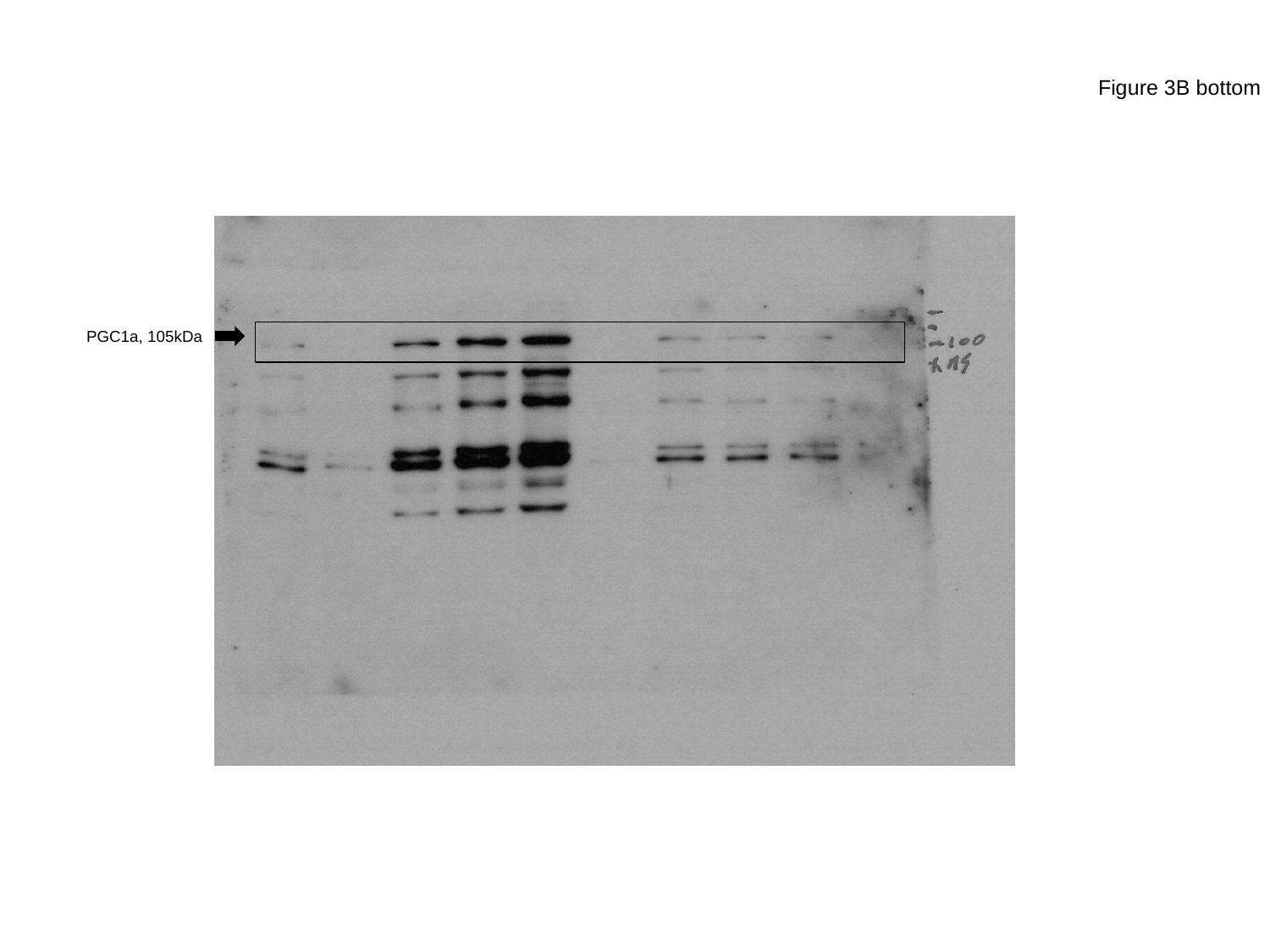

Figure 3B bottom
PGC1a, 105kDa

## Slide 5
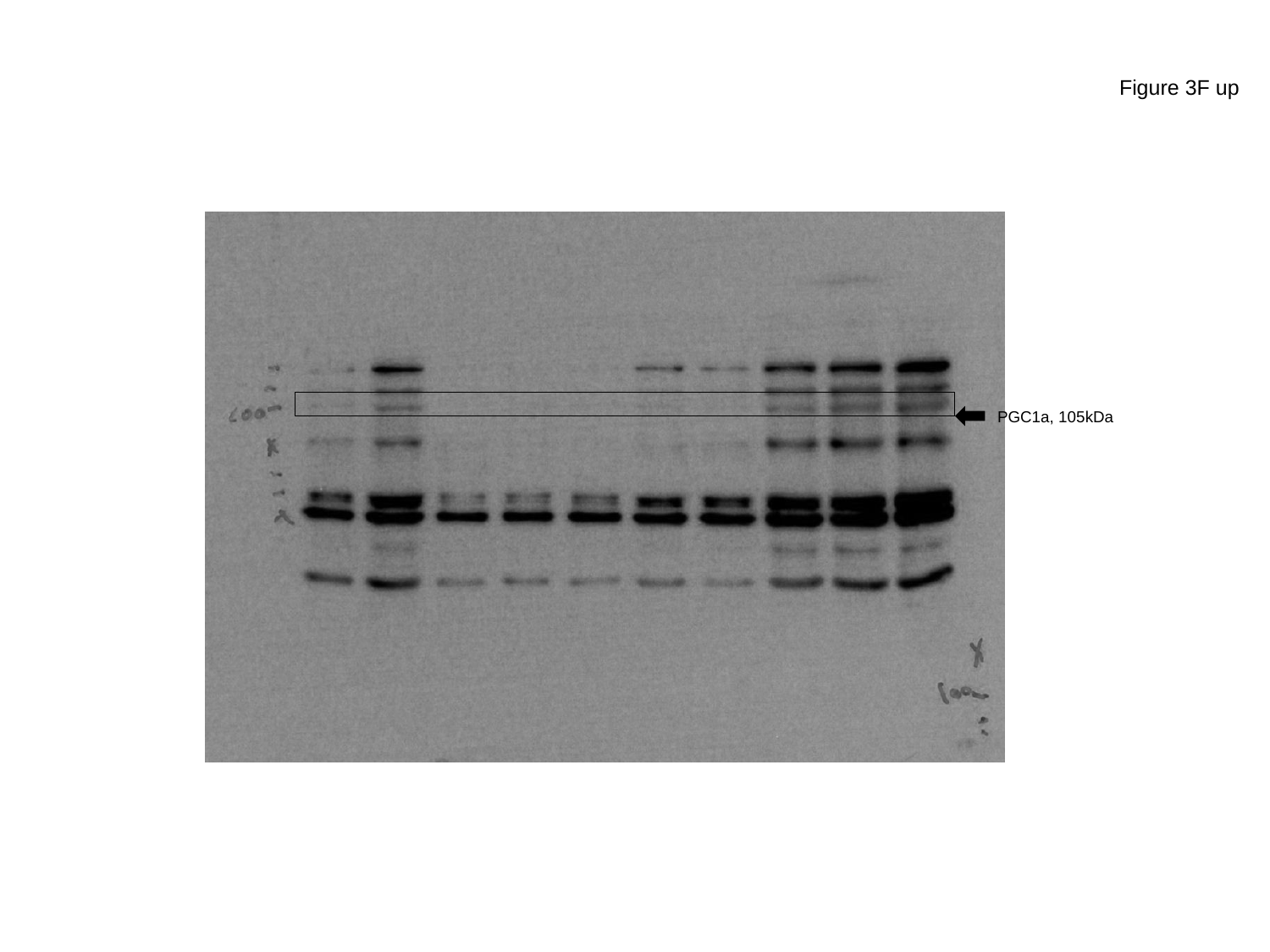

Figure 3F up
PGC1a, 105kDa

## Slide 6
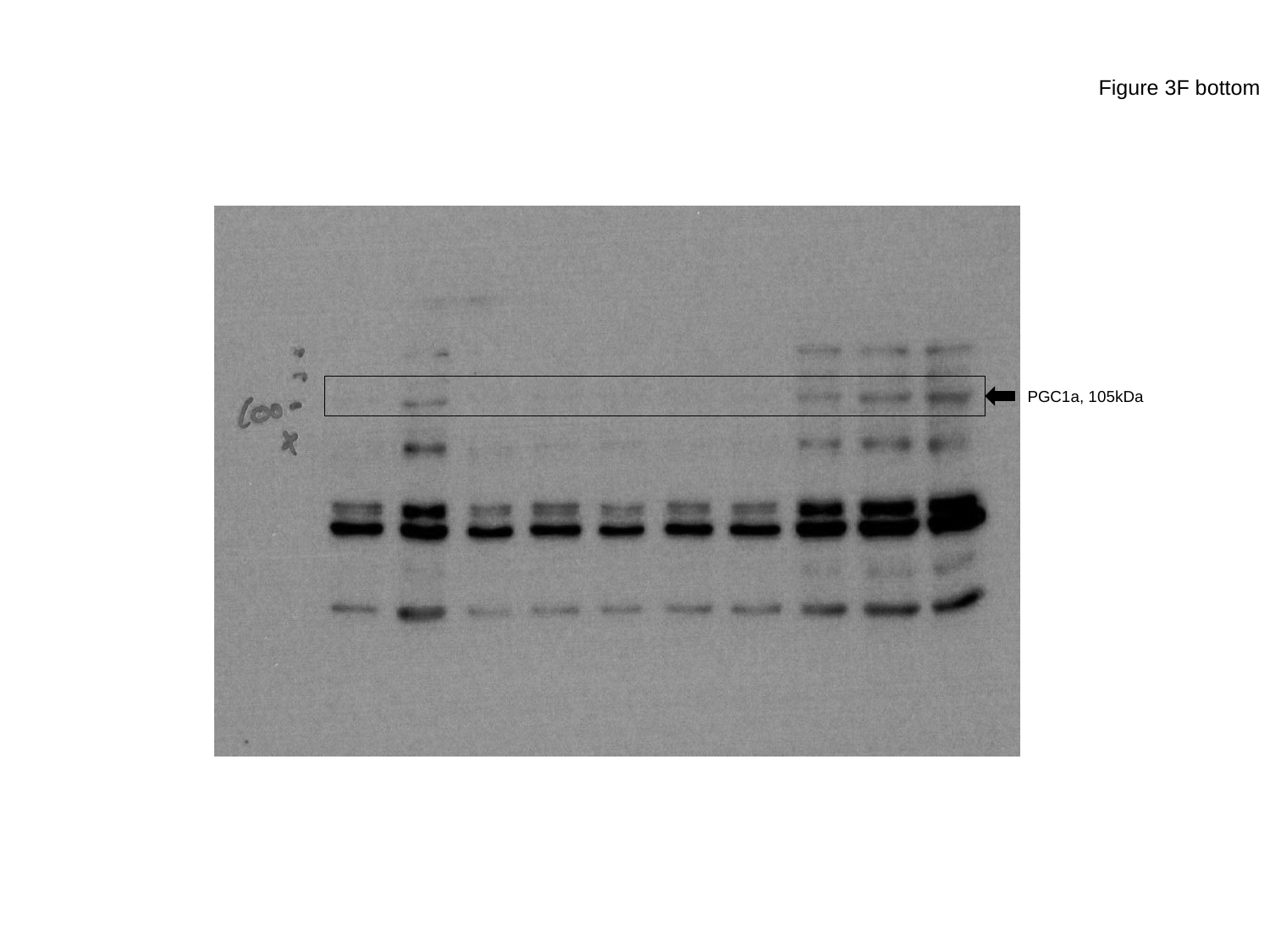

Figure 3F bottom
PGC1a, 105kDa

## Slide 7
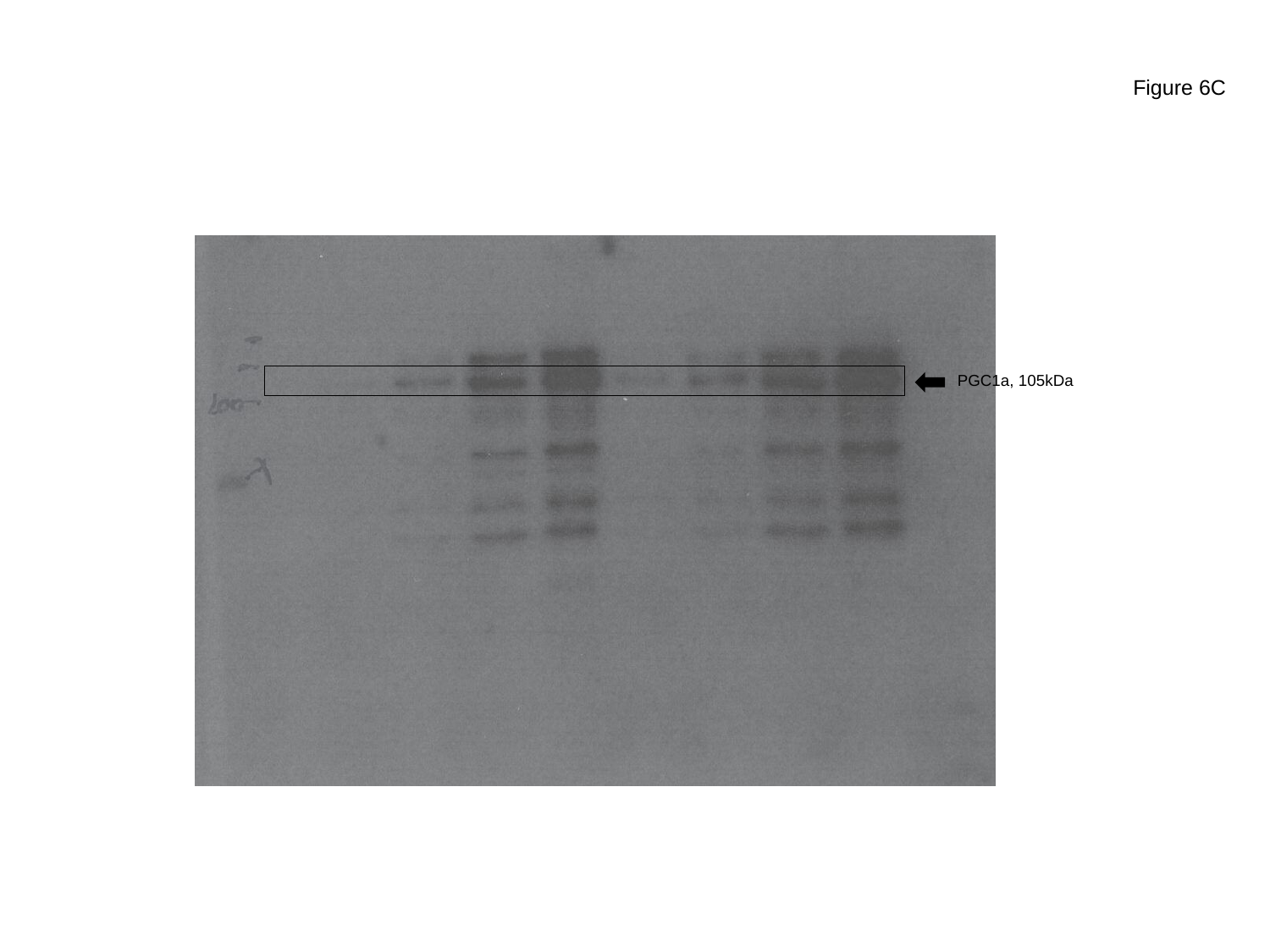

Figure 6C
PGC1a, 105kDa

## Slide 8
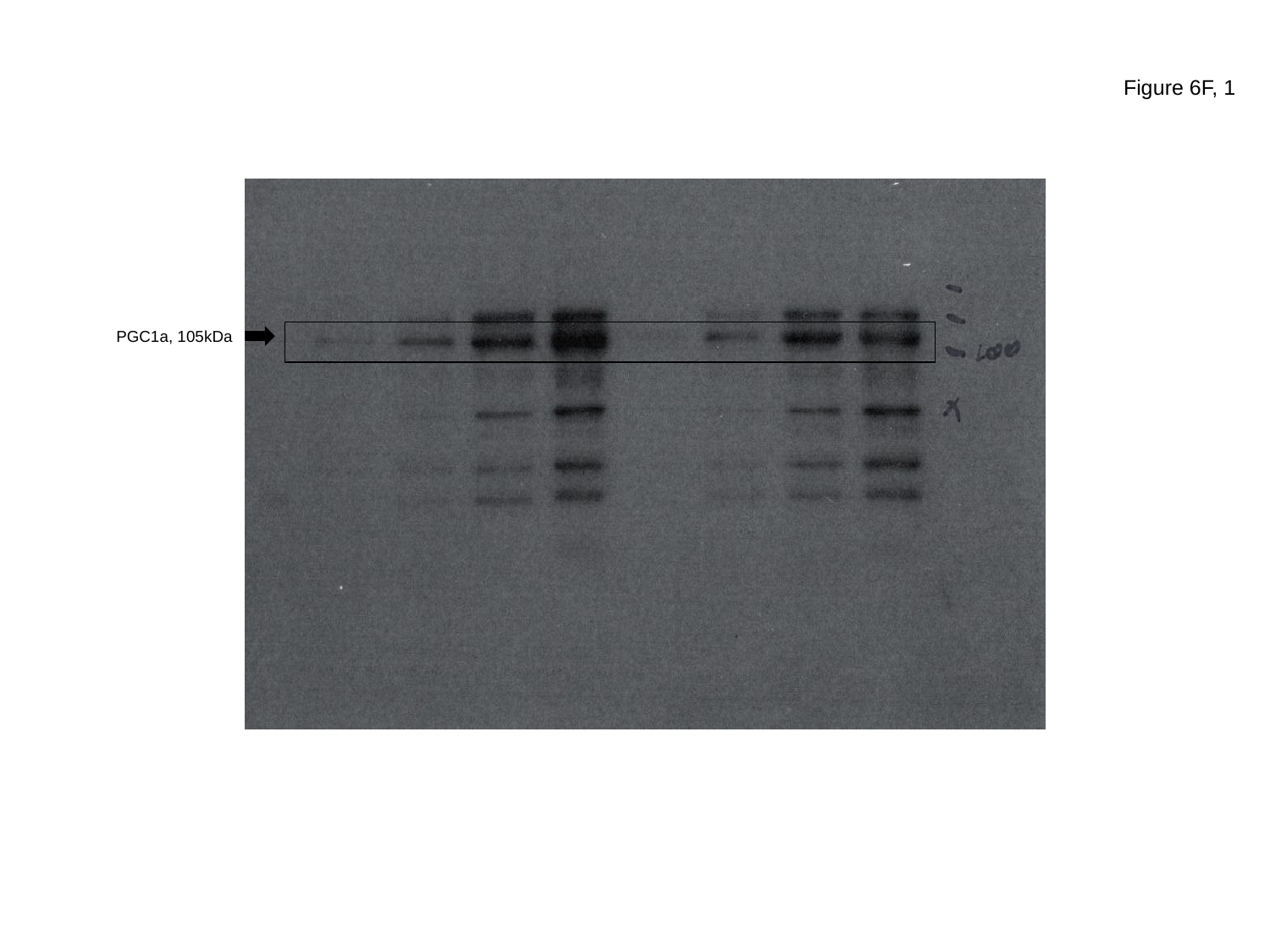

Figure 6F, 1
PGC1a, 105kDa

## Slide 9
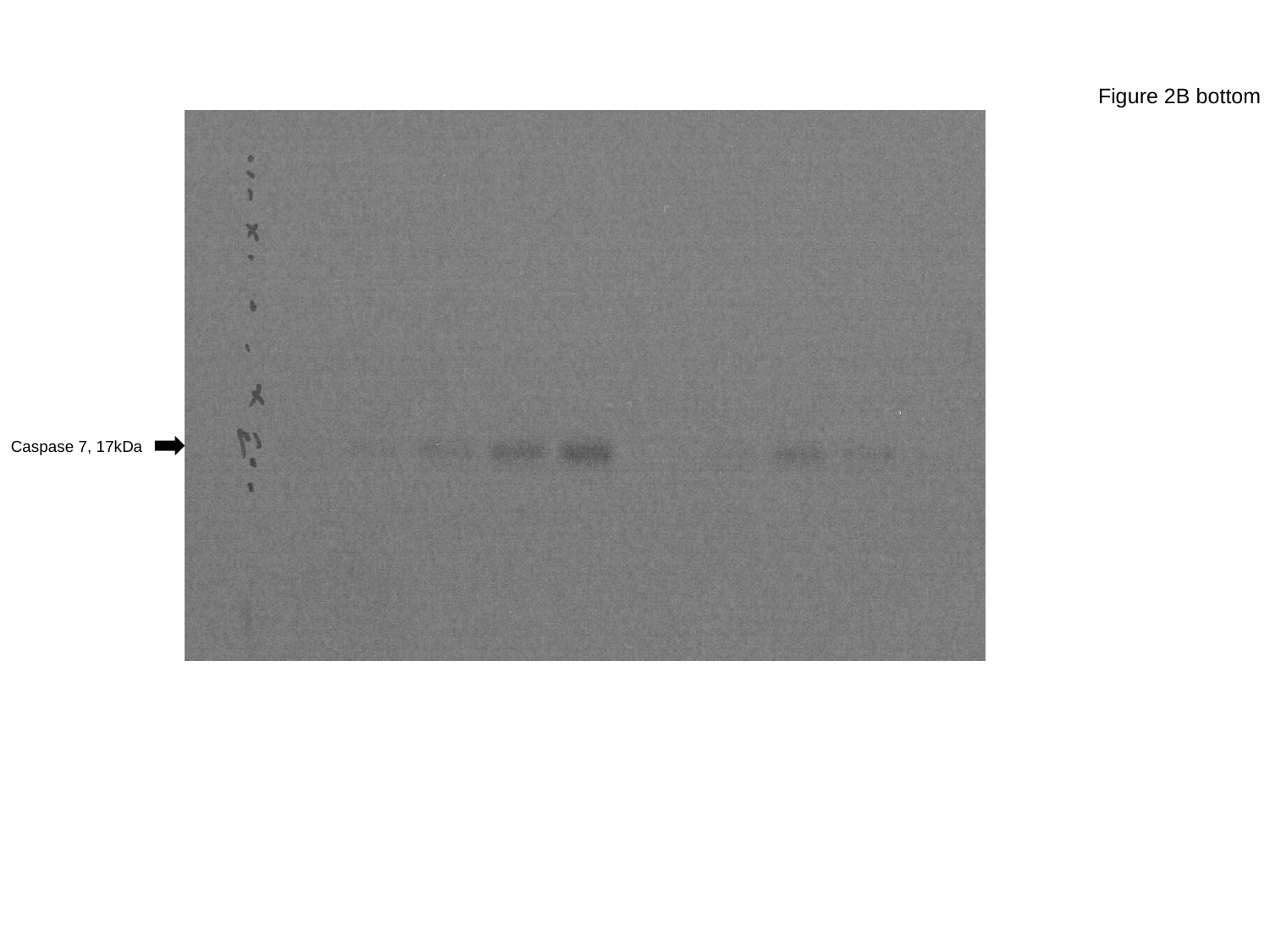

Figure 2B bottom
Caspase 7, 17kDa

## Slide 10
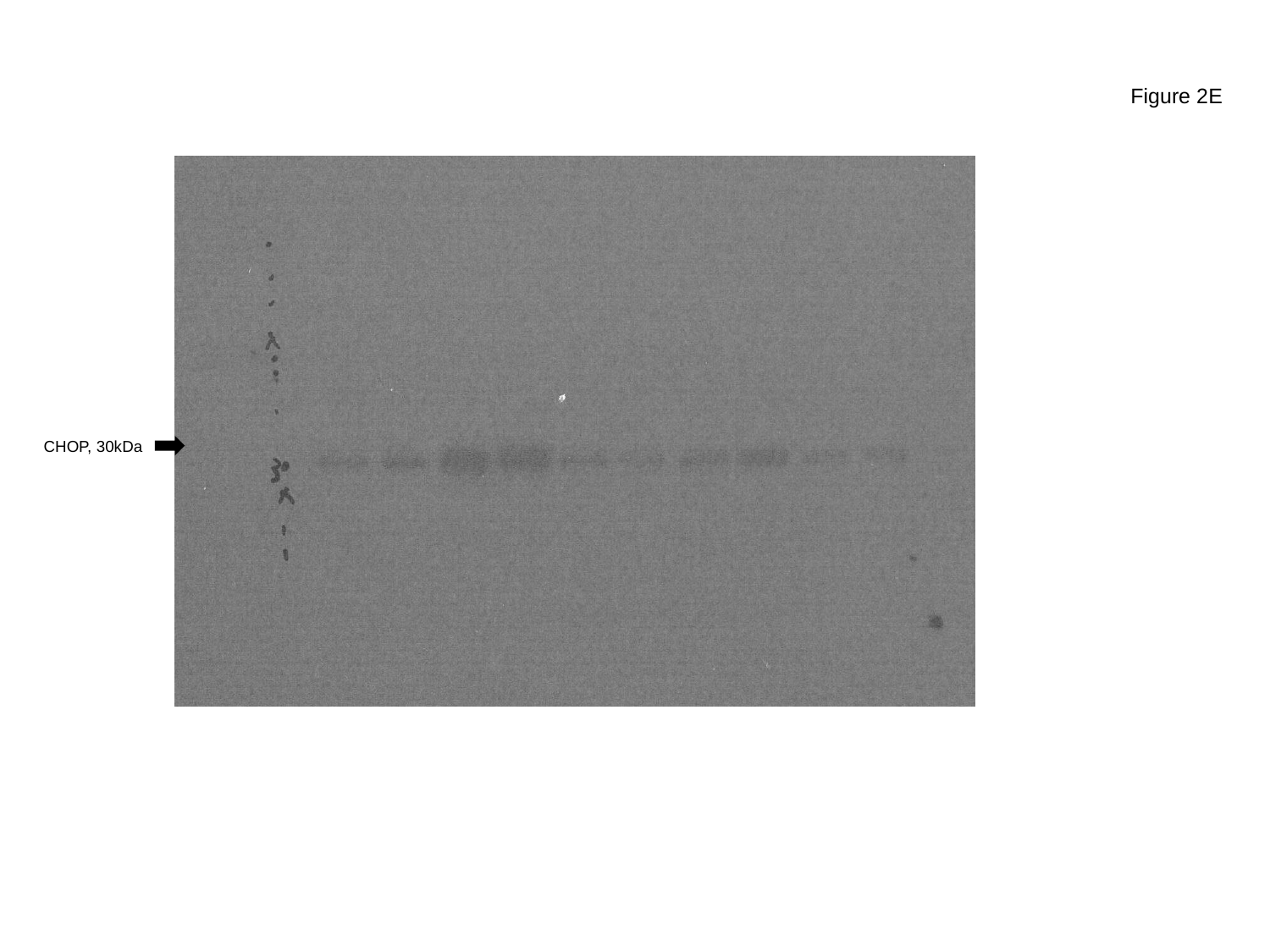

Figure 2E
CHOP, 30kDa

## Slide 11
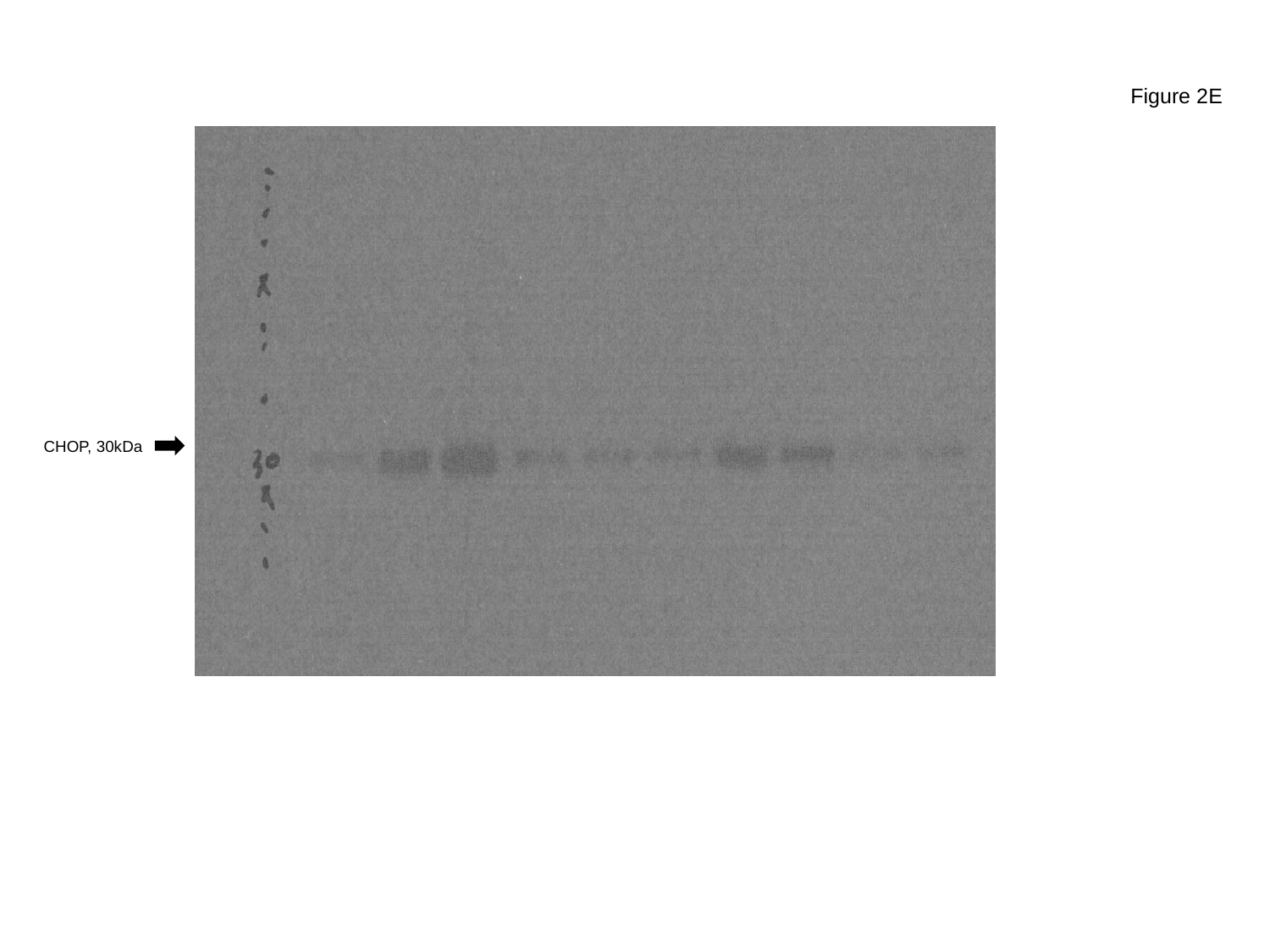

Figure 2E
CHOP, 30kDa

## Slide 12
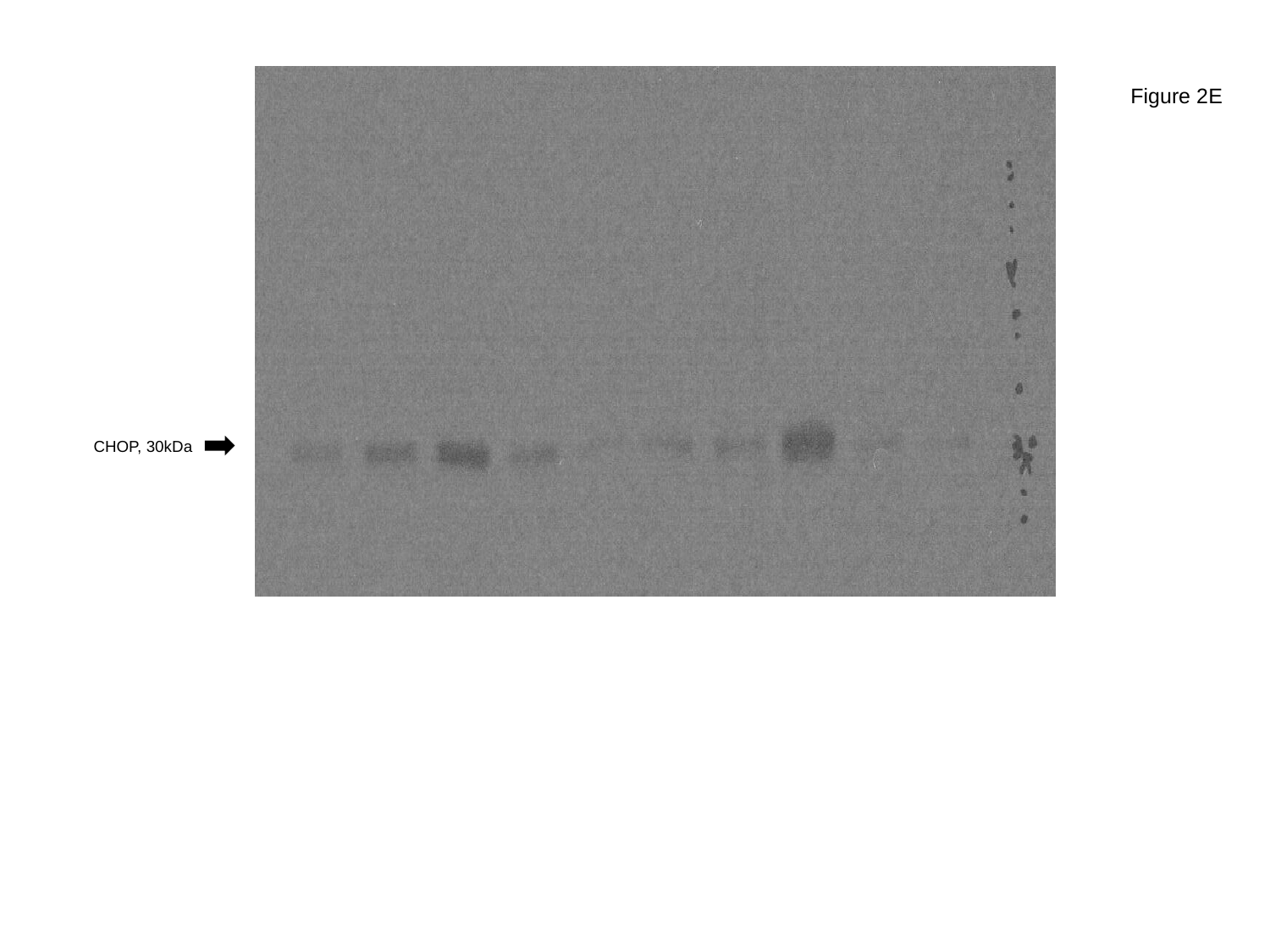

Figure 2E
CHOP, 30kDa

## Slide 13
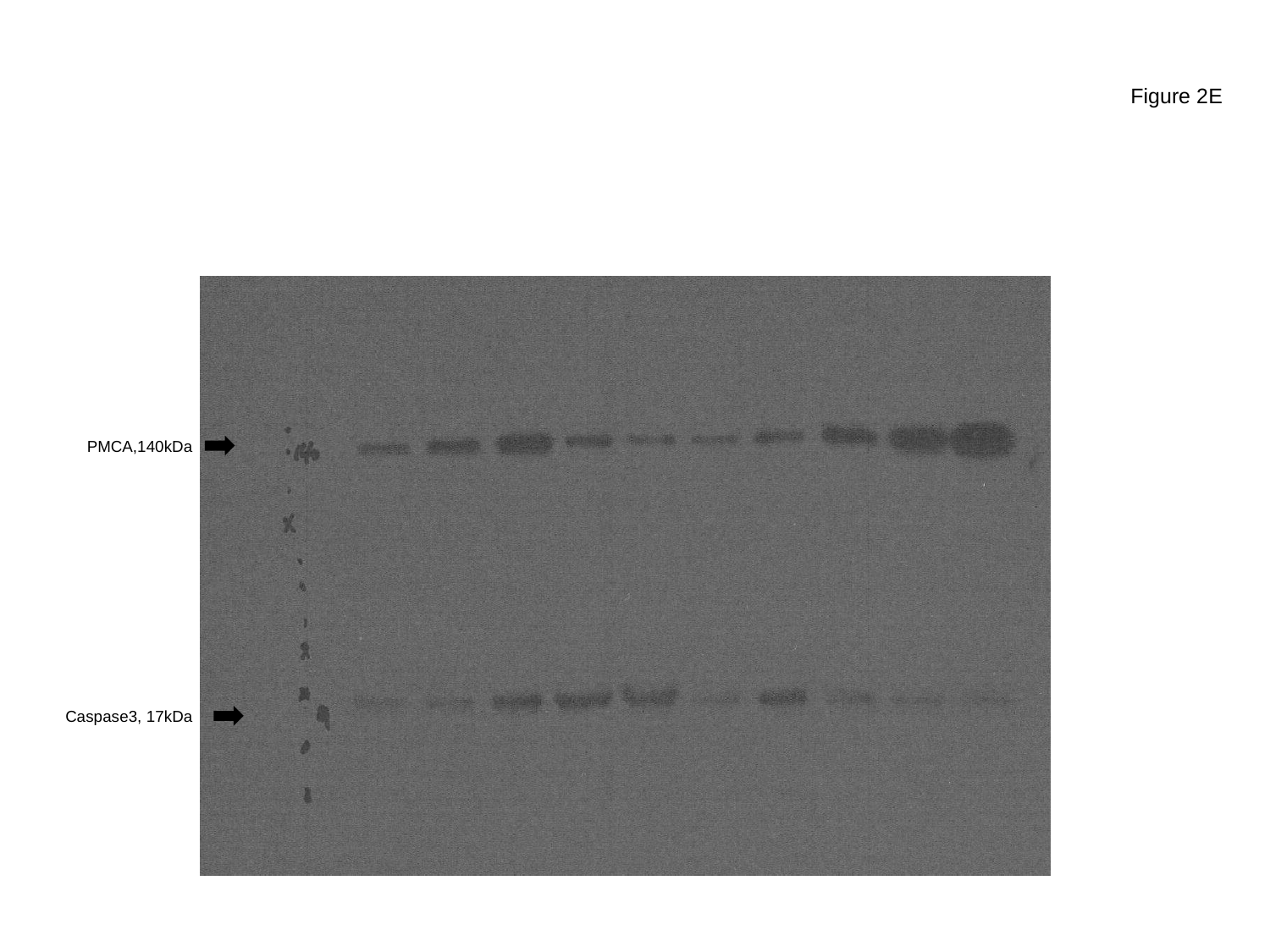

Figure 2E
PMCA,140kDa
Caspase3, 17kDa

## Slide 14
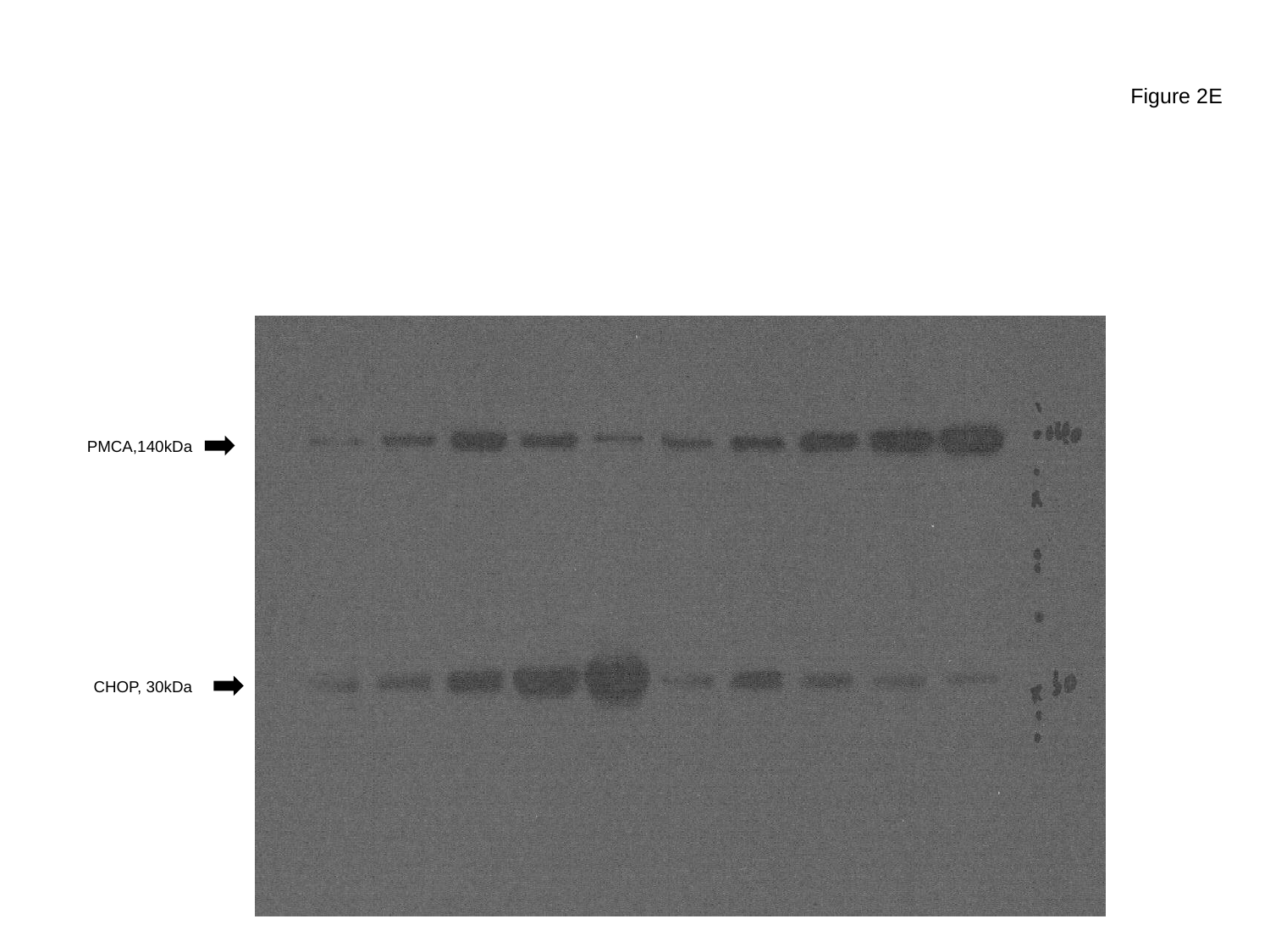

Figure 2E
PMCA,140kDa
CHOP, 30kDa

## Slide 15
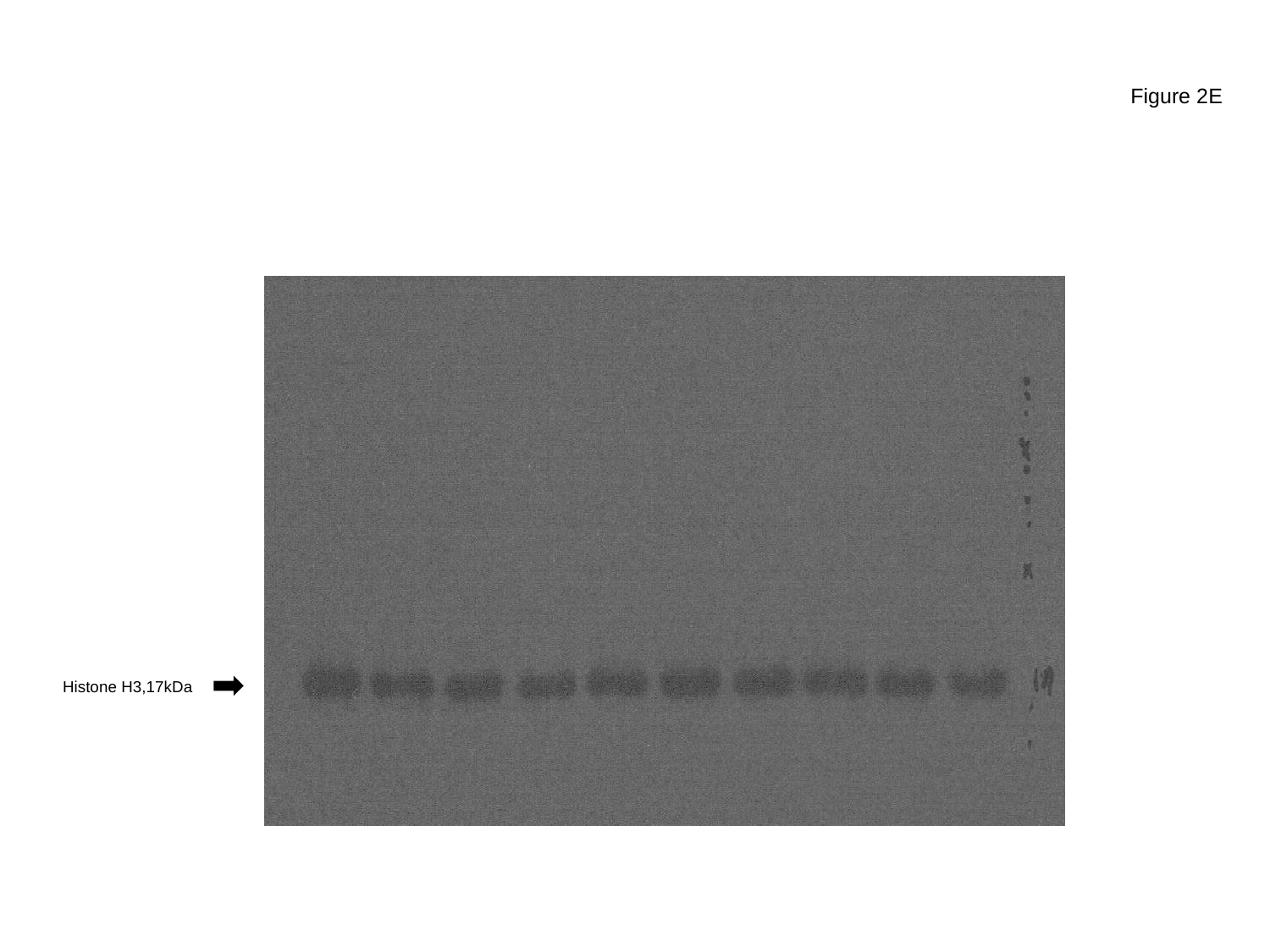

Figure 2E
Histone H3,17kDa

## Slide 16
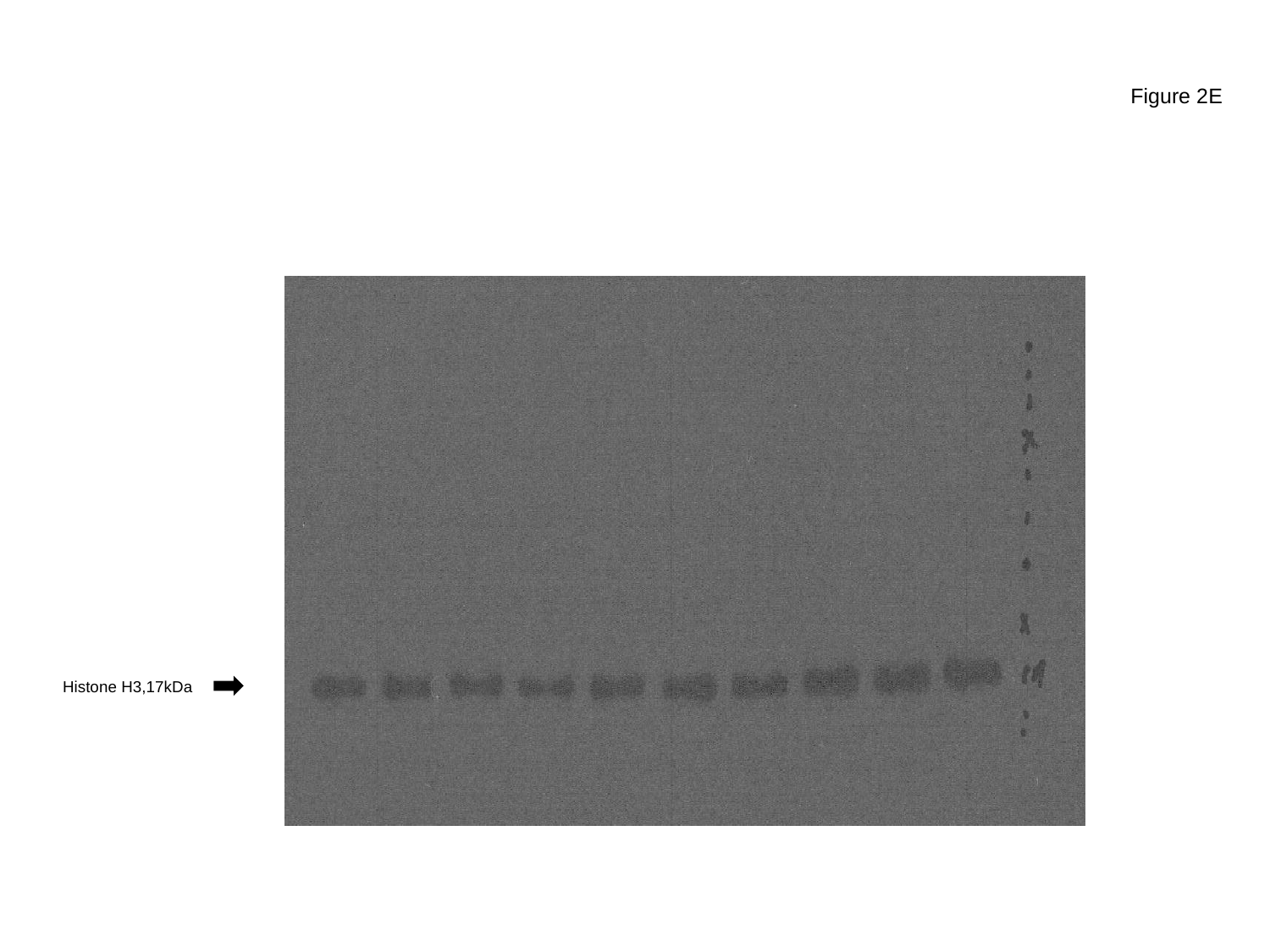

Figure 2E
Histone H3,17kDa

## Slide 17
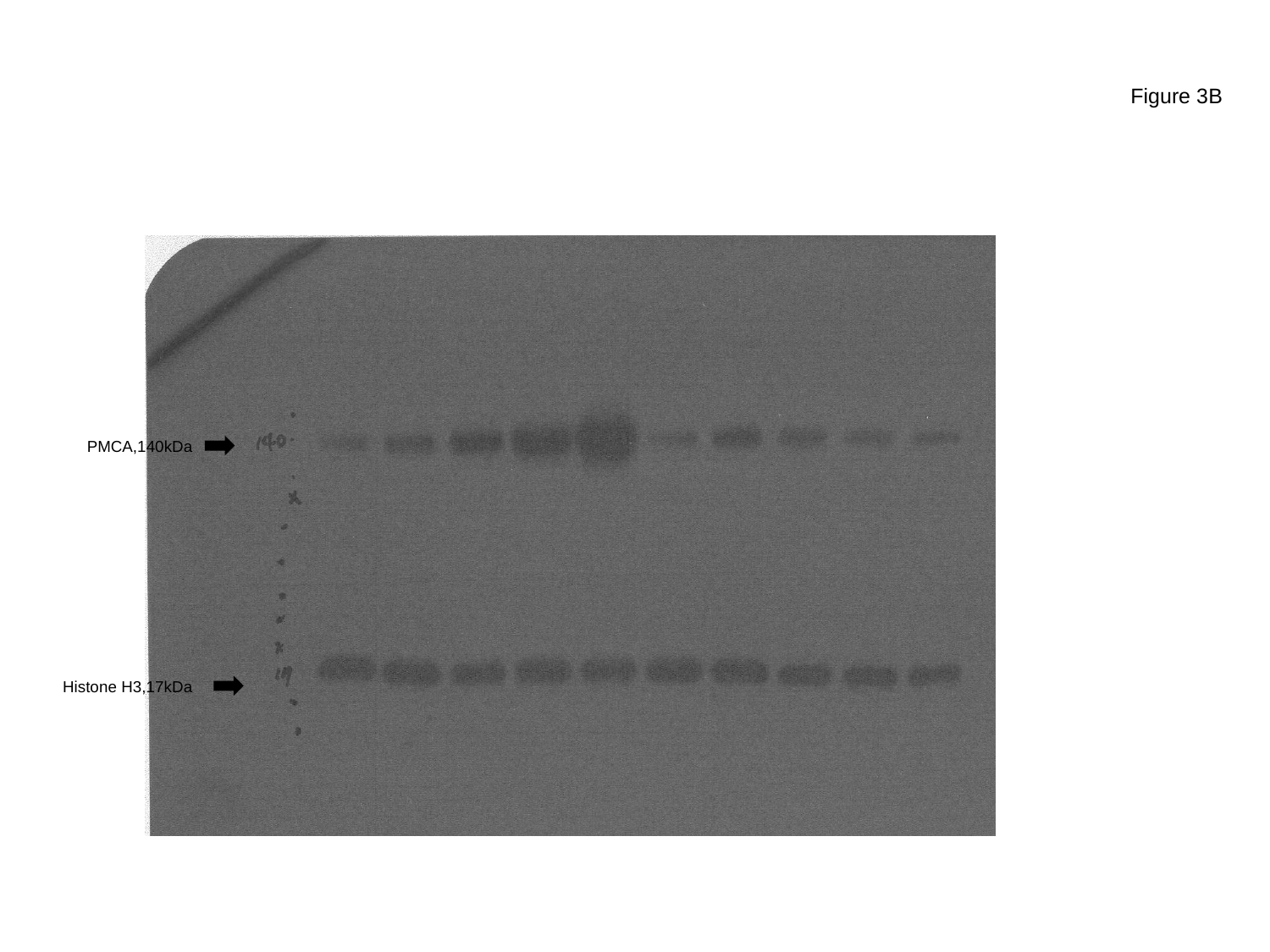

Figure 3B
PMCA,140kDa
Histone H3,17kDa

## Slide 18
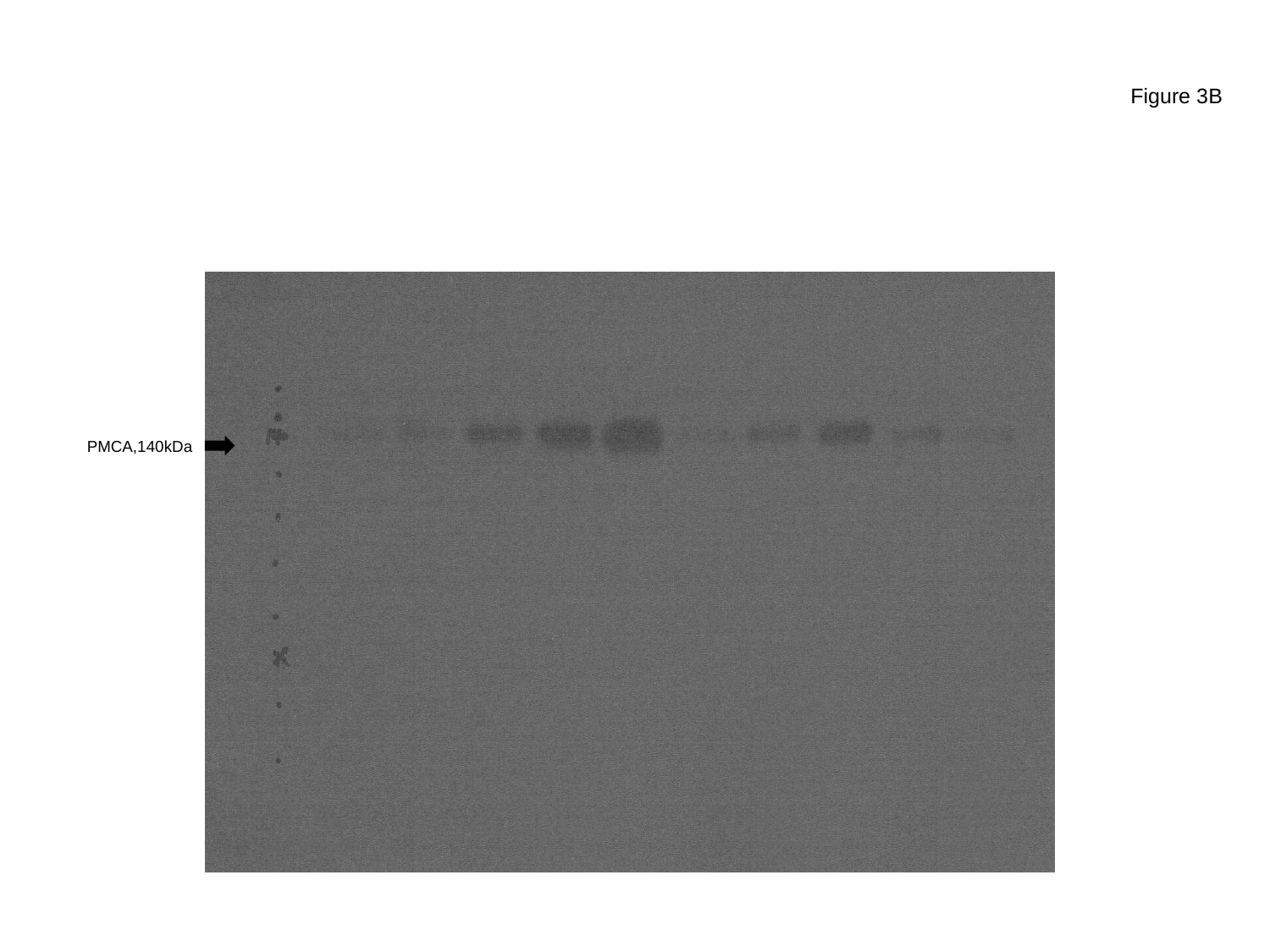

Figure 3B
PMCA,140kDa

## Slide 19
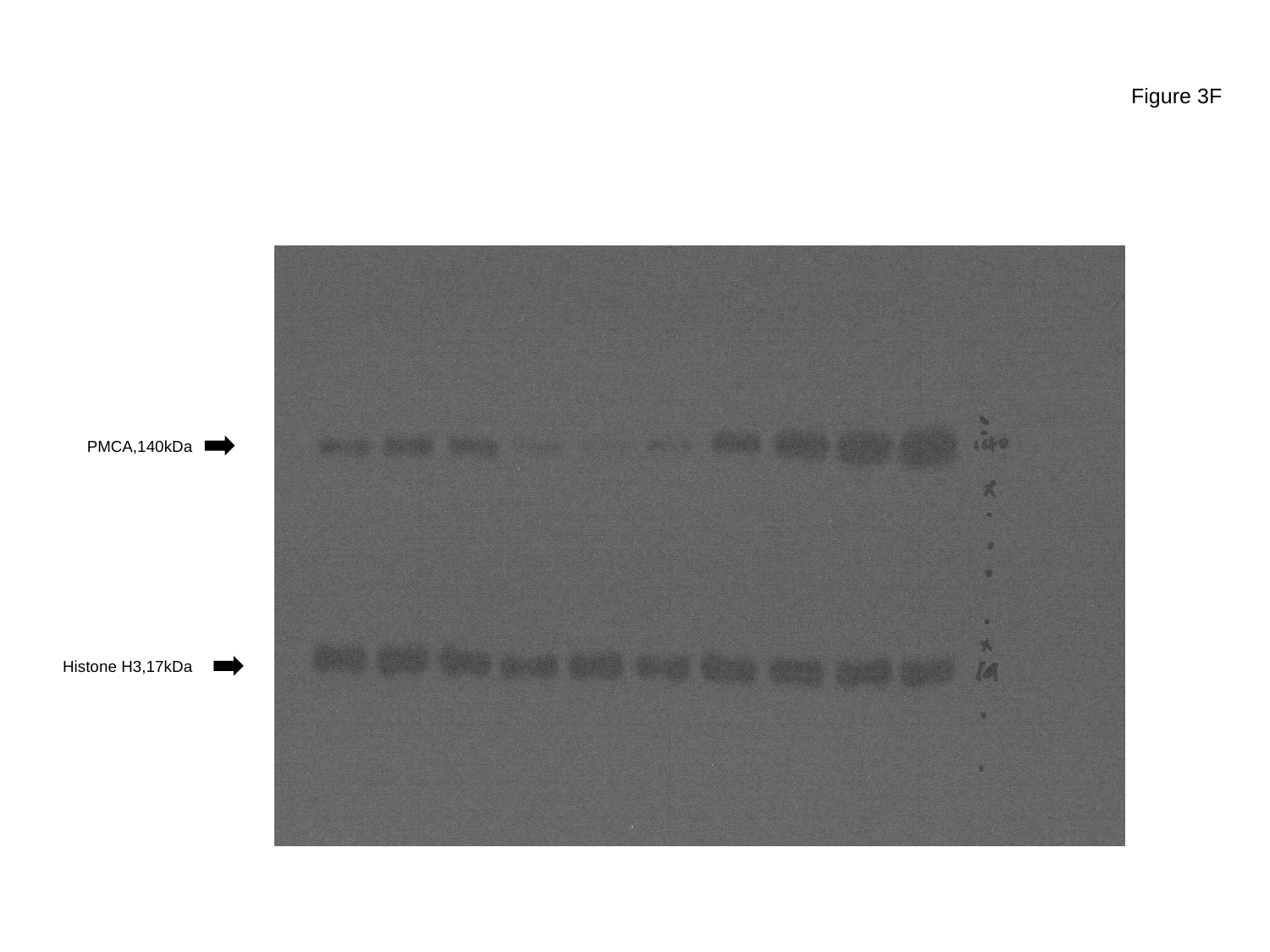

Figure 3F
PMCA,140kDa
Histone H3,17kDa

## Slide 20
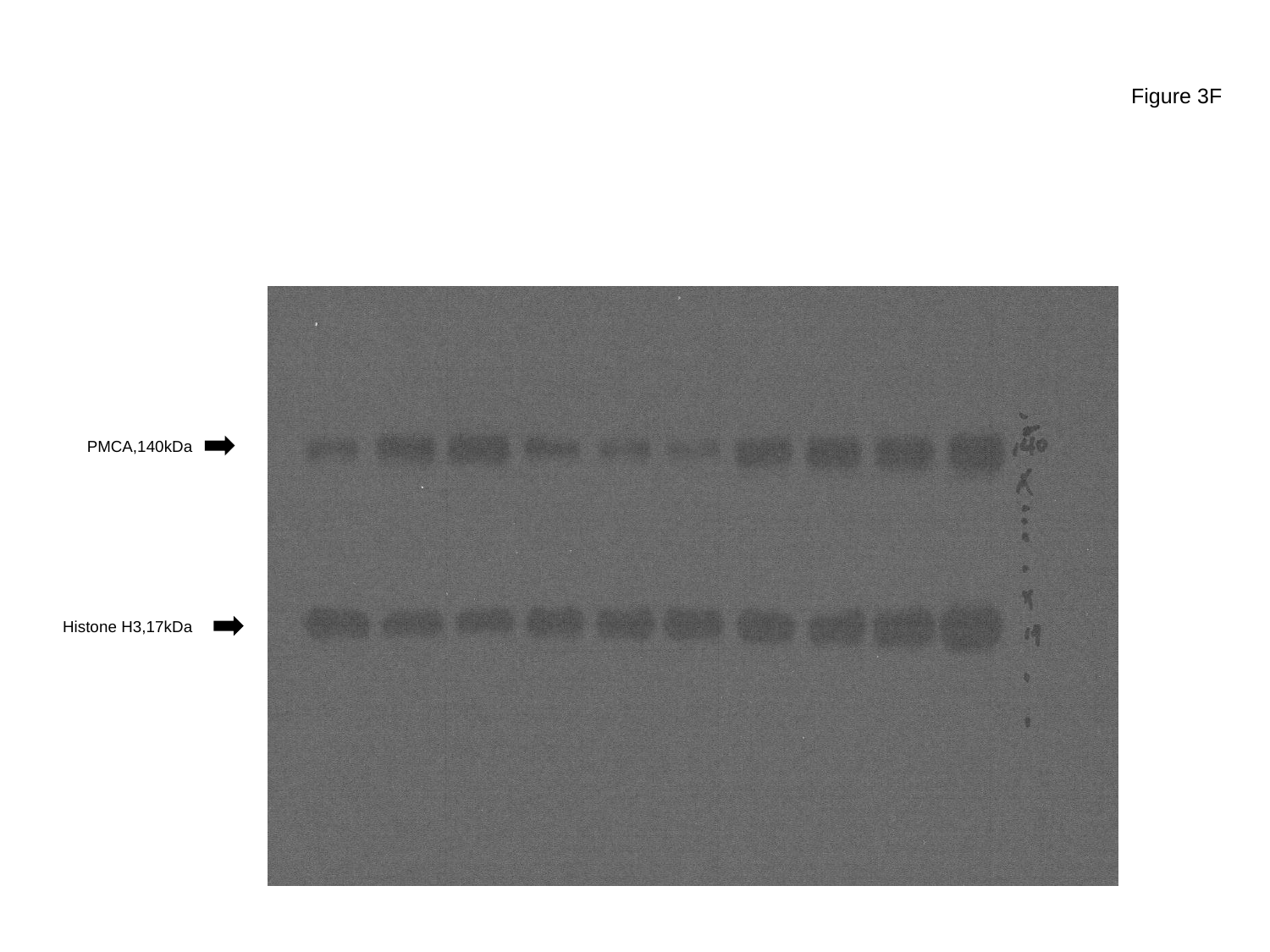

Figure 3F
PMCA,140kDa
Histone H3,17kDa

## Slide 21
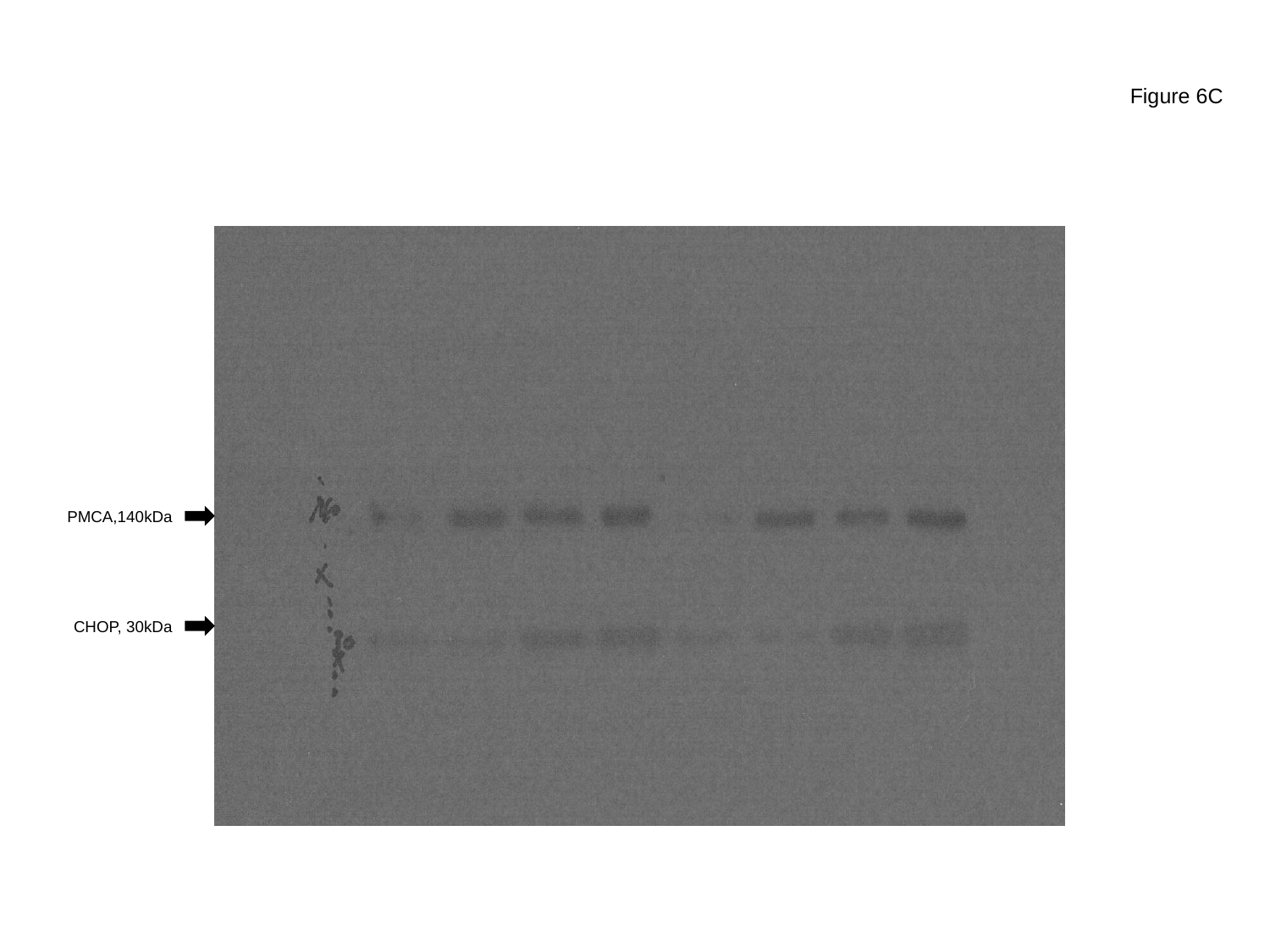

Figure 6C
PMCA,140kDa
CHOP, 30kDa

## Slide 22
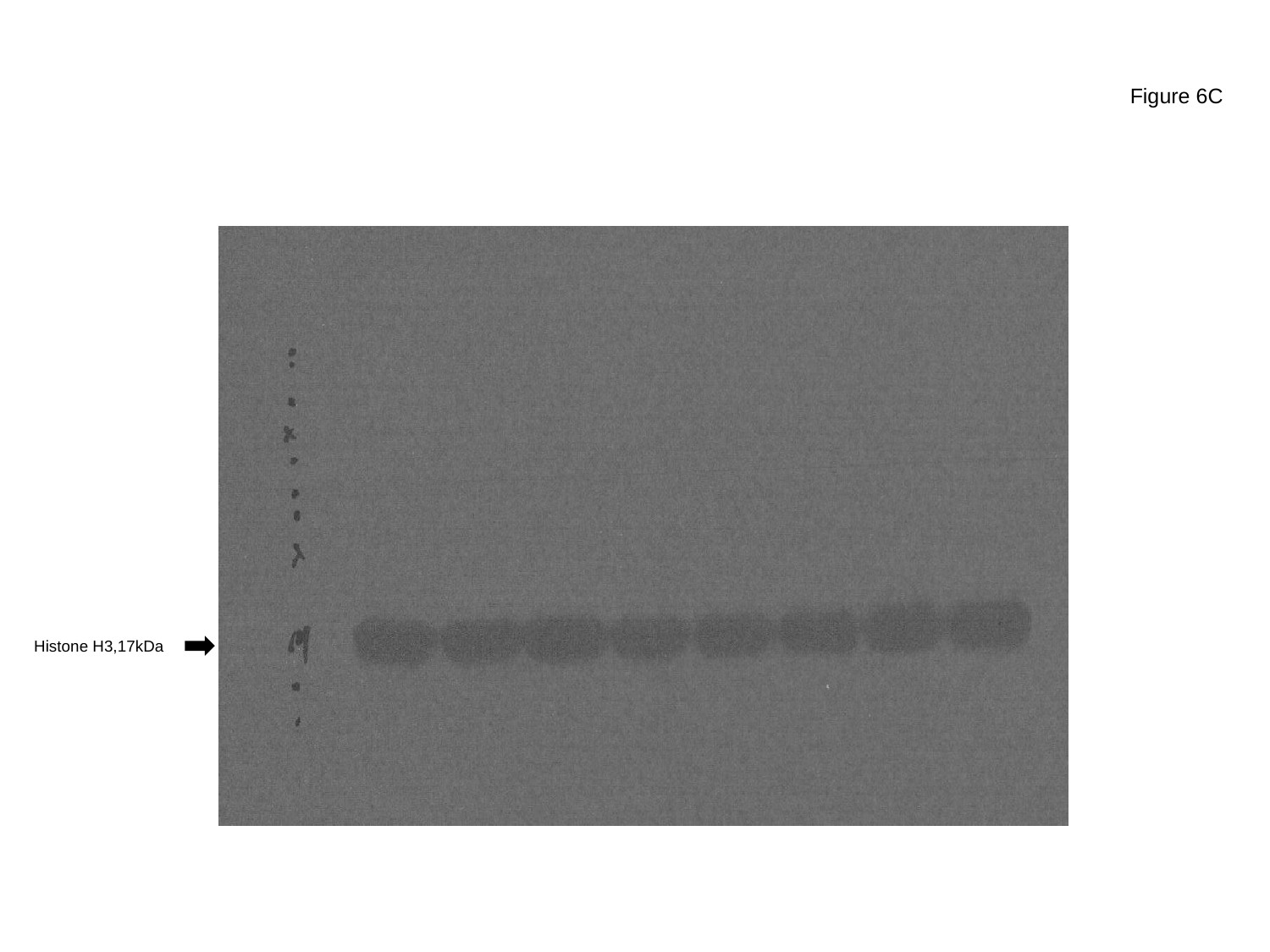

Figure 6C
Histone H3,17kDa

## Slide 23
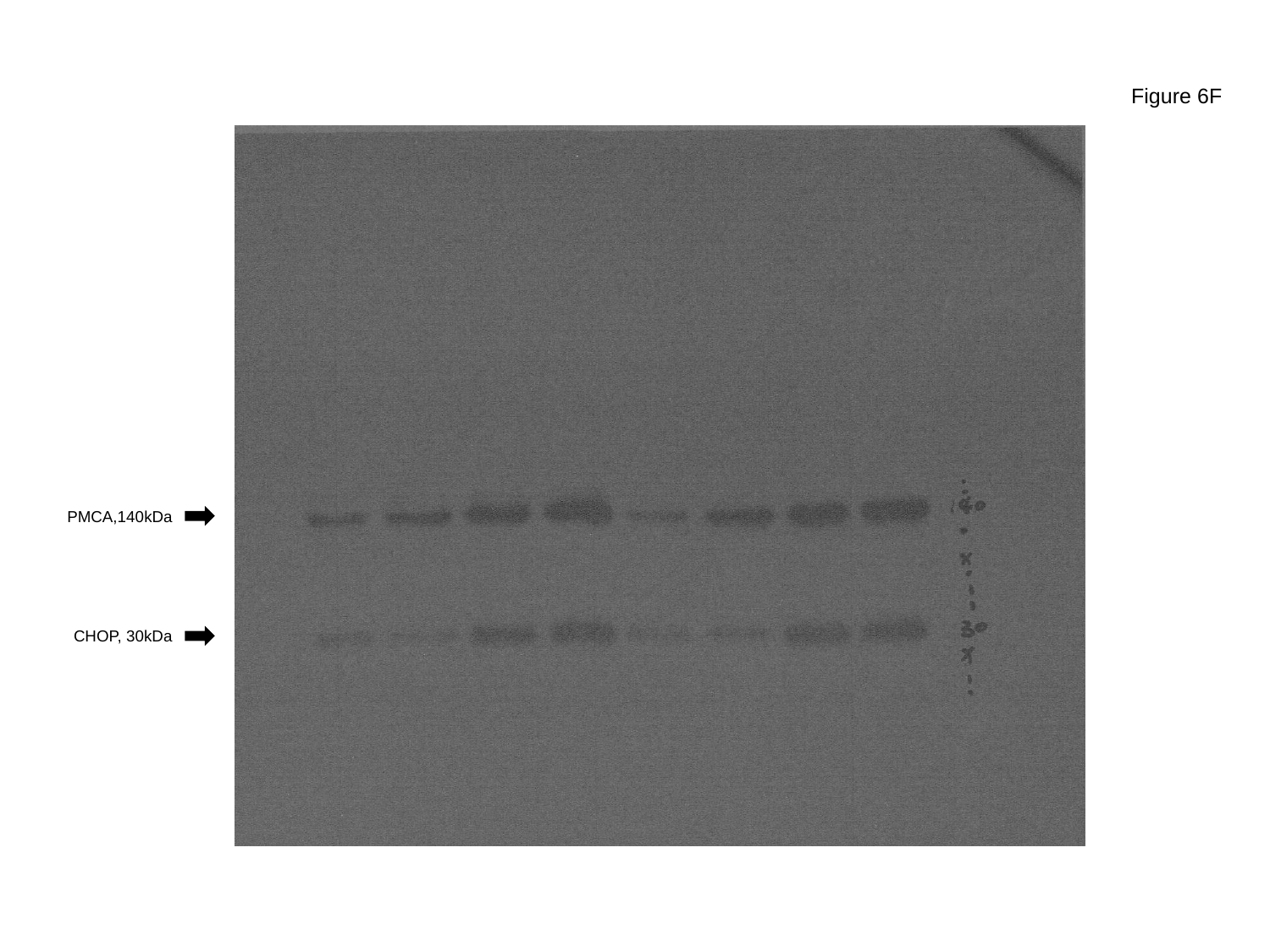

Figure 6F
PMCA,140kDa
CHOP, 30kDa
